# Tribochemistry of adaptive integrated interfaces at boundary lubricated contacts

**DOI:** 10.1038/s41598-017-09879-7

**Published:** 2017-08-30

**Authors:** Shanhong Wan, Anh Kiet Tieu, Yana Xia, Liping Wang, Dongshan Li, Guangan Zhang, Hongtao Zhu, Bach H. Tran, David R. G. Mitchell

**Affiliations:** 1 Faculty of Engineering and Information Sciences, University of Wollongong, Wollongong, 2500 Australia; 2 State Key Laboratory of Solid Lubrication, Lanzhou Institute of Chemical Physics, Chinese Academy of Sciences, Lanzhou, 730000 China; 3 Electron Microscopy Centre, University of Wollongong, Wollongong, NSW 2522 Australia

## Abstract

Understanding how an adaptive integrated interface between lubricant additives and solid contacts works will enable improving the wear and friction of moving engine components. This work represents the comprehensive characterization of compositional and structural orientation at the sliding interface from the perspective of surface/interface tribochemistry. The integrated interface of a lubricant additive-solid resulting from the friction testing of Graphite-like carbon (GLC) and PVD-CrN coated rings sliding against cast iron under boundary lubrication was studied. The results indicate that in the case of the CrN/cast iron pair the antiwear and friction behavior were very strongly dependent upon lubricant. In contrast, the tribology of the GLC surface showed a much lower dependence on lubrication. In order to identify the compounds and their distribution across the interface, x-ray microanalysis phase mapping was innovatively applied and the principle of hard and soft acids and bases (HSAB) to understand the behaviour. Phase mapping clearly showed the hierarchical interface of the zinc-iron polyphosphate tribofilm for various sliding pairs and different sliding durations. This interface structure formed between lubricant additives and the sliding surfaces adapts to the sliding conditions – the term adaptive interface. The current results help explain the tribology of these sliding components in engine.

## Introduction

Achieving the optimum lubrication and friction in automotive applications, represents a potential for reduced energy consumption and therefore reduced emissions. The moving engine components frequently operate under boundary lubrication, and oil additives work by forming boundary tribofilms between the rubbing parts. The effective interactions that are based on lubricant additives-solid contacts determine the tribological performance. These in turn depend upon the physics and chemistry of the boundary film. Graphite-like amorphous carbon (DLC)^1, 2^ and chromium nitride (CrN)^3, 4^ have been applied onto engine components to minimize friction and wear. However, they are non-ferrous surfaces. Currently, the oil formulation is optimised for ferrous surfaces. Although the utilization of non-ferrous surfaces results in a significant decrease in friction and wear^5^, a concern is that the application of non-ferrous coating may neutralise the benefits of using additives in engine oil. As described by Erdemir^6^, non-ferrous surfaces and lubricants could be regarded as a single system. The fundamental tribochemistry at the adaptive interface between the boundary layer lubricant and non-ferrous solids need to be better understood.

Intensive work has been carried out to evaluate the interactions between non-ferrous coatings and lubricant additives with regard to wear and friction plus the interfacial chemistry of the tribofilm, e.g. the CrN/steel and DLC/steel contacts^7–10^. For CrN/steel contacts, additive concentrations and additive types as well as the contacting conditions determine the friction and wear properties. Anti-wear additives confer complete protection of a CrN surface, while friction modifiers result in excessively high wear^11^. Although tribofilms readily form on CrN, increasing friction occurs as well as an acceleration of wear on the opposing surface. In contrast, DLC/steel contacts result in superior tribological performance even in dry sliding conditions^1, 2, 7–10^. This is reported to be due to a nano-scale tribofilm with a predominantly sp^2^-type carbon surface which confers self-lubricating capability. However, when lubricant is present, the interaction between lubricant additives and the DLC surface is essential but contradictory^8–16^. Under boundary lubrication, some researchers cannot find the tribofilm on DLC surface in the presence of oil additives, while some argue that tribofilm growth onto DLC surface does occur^15^. Spikes *et al*.^8^ and Podgornik *et al*.^9^ reported that greater wear resulted on a steel surface slid against DLC when antiwear additives were present in the lubricants. This detrimental effect was due to the unexpected competition between the Zinc dialkylthiophosphate (ZDDP)-produced tribofilm and a DLC-induced carbon transfer onto a steel ball^8^. Qu *et al*.^10^ showed the increasing wear rate on the steel opposing DLC was correlated with the DLC-catalysed, high rate tribochemical reactions with organophosphate additives. These studies highlight that the tribofilm formation and interactions between additives and rubbing contacts are taking place, but they do not provide a comprehensive understanding of the working mechanics of the integrated interface. There is still much work which needs to be done in order to understand how non-ferrous surfaces and tribochemically reactive film work together in order to optimise the tribology.

The formation and removal kinetics of the tribofilm influences the tribological responses. Extensive surface and interfacial characterizations have been carried out^7, 14, 16, 17^. Gosvami *et al*.^16^ evaluated the growth and properties of a ZDDP-derived tribofilm by an *in-situ* approach using atomic force microscopy. The growth rate of the tribofilm increases exponentially with both applied compressive stress and temperature; a thermally-activated, stress-assisted model is involved with regard to the growth rate of the tribofilm^16^. In contrast, on the basis of first-principle atomistic calculations, Mosey *et al*.^17^ proposed that tribofilm formation is from the contribution of contact -induced cross-linking of zinc phosphate molecules from a thermal or catalytic decomposition of ZDDP. Previous research work mostly concentrated on the elemental composition and hierarchical structure analysis of ZDDP-derived tribofilms. Since formulated oil lubricant always involves complex constituents, the evolution of chemical compounds and their orientation across the tribological interface has not been reported to date.

In this work, we investigate two types of non-ferrous coating (PVD-CrN and PVD-CrN + GLC^18, 19^), and evaluate the cooperative action of non-ferrous surfaces and lubricant additives by friction tests. In order to illustrate tribofilm kinetics, the influence of lubricant quantity was evaluated to understand sliding under conditions ranging from fully lubricated through to oil-starved lubrication, where the lubricating condition became increasingly scarce over a small quantity of lubricant in this work. Real piston rings with non-ferrous coatings and cylinder liners have been selected for this investigation, combined with commercially formulated oil. This work provides a comprehensive understanding of the tribochemistry across integrated interfaces of non-ferrous surfaces and lubricant media. In particular, the friction-induced pattern of compound formation and their disposition with respect to the surface is revealed.

## Results

### Friction and wear results

**Figure 1:**
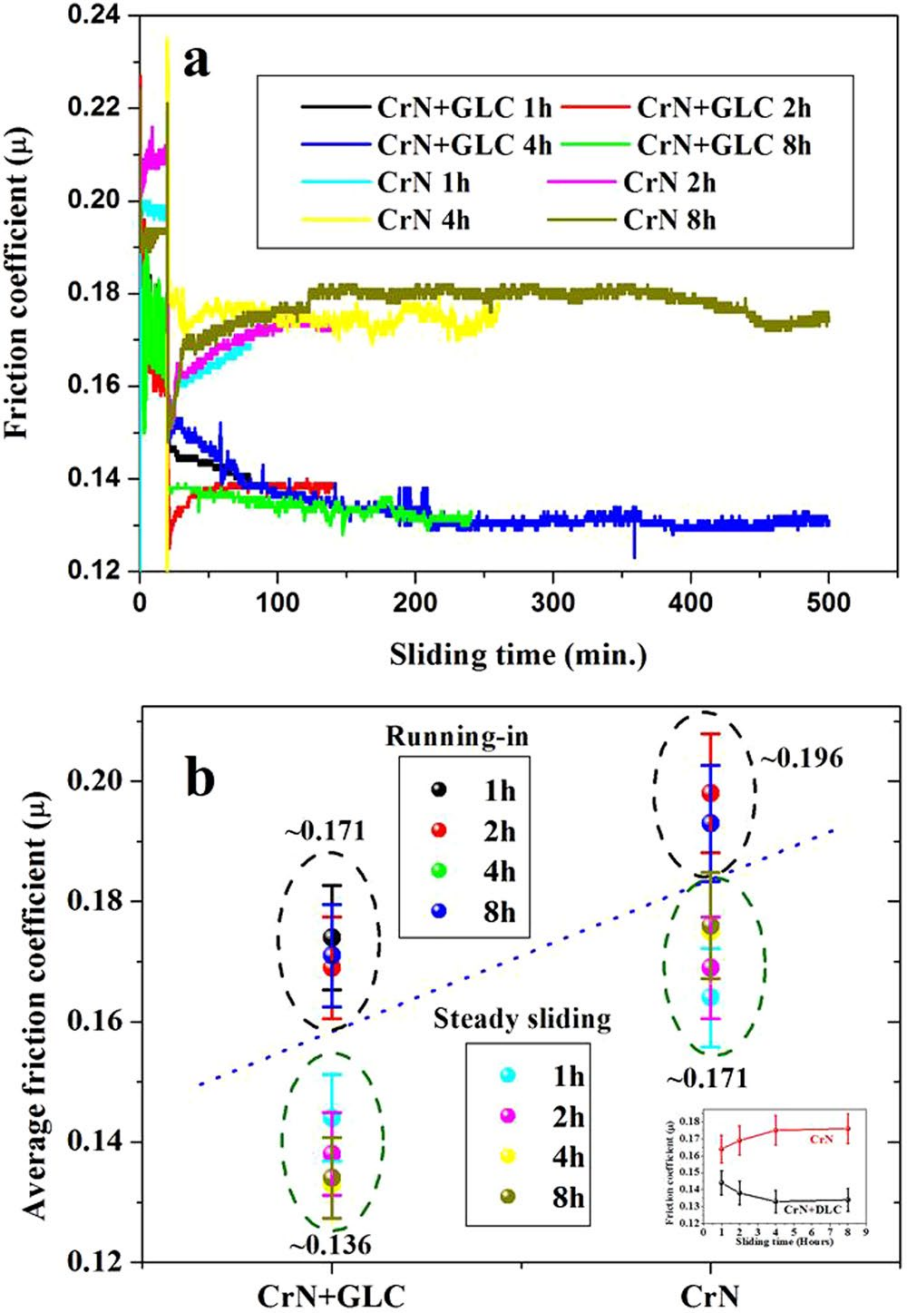
(**a**) Variation of friction coefficient with sliding time in oil lubrication, (**b**) Average friction coefficients of CrN/steel and CrN + GLC/steel pairs in running-in and steady-sliding periods. The insert in Fig. 1b displayed the trends of average friction coefficients at different sliding periods.

The friction and wear characteristics of CrN/cast iron and CrN + GLC/cast iron tribo pairs were evaluated under an increasingly oil-starved condition, each rubbing pair was subjected to multiple test durations of 1, 2, 4, and 8 hours. Figure 1a shows the variation of friction coefficients for the CrN/cast iron and CrN + GLC/cast iron pairs for various sliding durations. The CrN + GLC/cast iron contact produced a constantly low friction value compared with that of the CrN/cast iron pair. At the initial running-in period, CrN + GLC/cast iron showed a friction coefficient of around 0.17, while CrN/steel contact had a value of 0.196 (Fig. 1b). With extended sliding, the friction increased gradually for CrN/cast iron, but the opposite was observed for CrN + GLC/cast iron pairs. The comparison of friction characteristics indicated that, CrN + GLC surface worked with lubricant additives effectively, minizing friction and stabilizing the sliding contacts.

**Figure 2:**
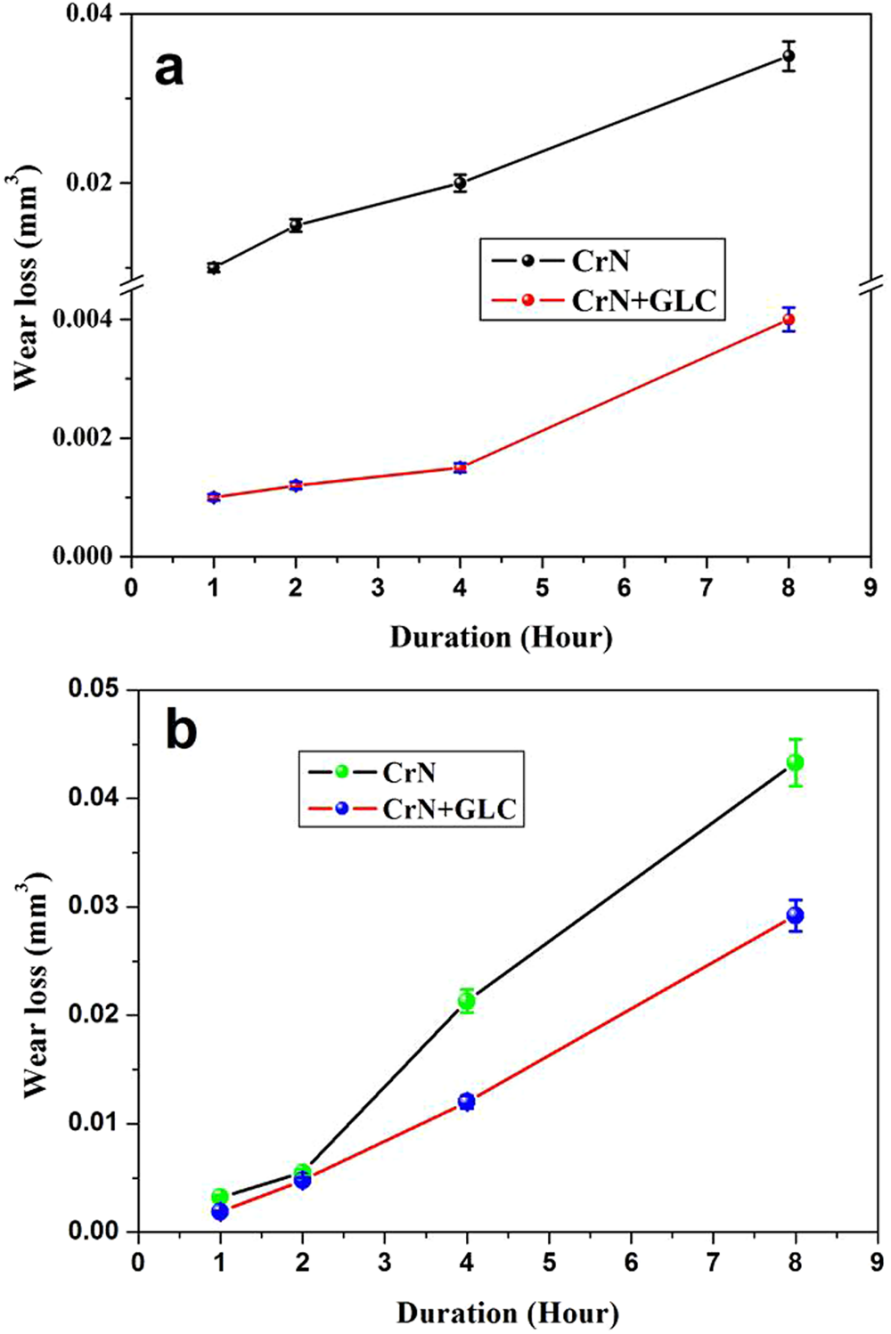
Wear loss of the coated rings (**a**) and the opposing cast iron samples (**b**) in oil lubrication.

The CrN coated ring showed that wear loss increased as a linear function of time (Fig. 2a). This suggested that a protective trib-film was not forming. In contrast, the GLC surface showed a much lower wear loss. As before, the wear loss increased with time, but it was always lower than that of CrN surface during rubbing by nearly one order of magnitude. When considering the wear on the cast iron cylinder liner, a linear rate of of wear with time could be observed on the cast iron samples regardless of the mating surfaces (Fig. 2b). The GLC coated CrN resulted in reduced wear loss on the corresponding cast iron a factor of approximately 2.

In this study, the lubricant was applied once only, prior to the start of the sliding experiment. The lubricant was thus gradually reduced with a simultaneous reduction of the additive concentration. Figures 1 and 2 well indicated that, the tribological performance of CrN/cast iron contact had the strong connection with the oil additives, while the application of CrN + GLC surface achieved a decreasing dependency on oil lubrication. Especially for the CrN + GLC/cast iron contacts, their enhanced tribological performance was inherently determined by the interfacial interaction of lubricant tribofilm and solid contacts during friction. Furthermore, there were different acting mechanisms occurring if amorphous carbon was involved between the moving contacts^8, 10^. A competition between ZDDP-derived tribofilm and transferring carbon has been hypothesized^8^, while carbon-catalyzed, high rate chemical reactions with additives was reported to be responsible for this increased wear^10^. This work would address the interacting mechanism of the adaptive integrated interface from the following surface and interface characterizations.

### Wear Morphologies

**Figure 3:**
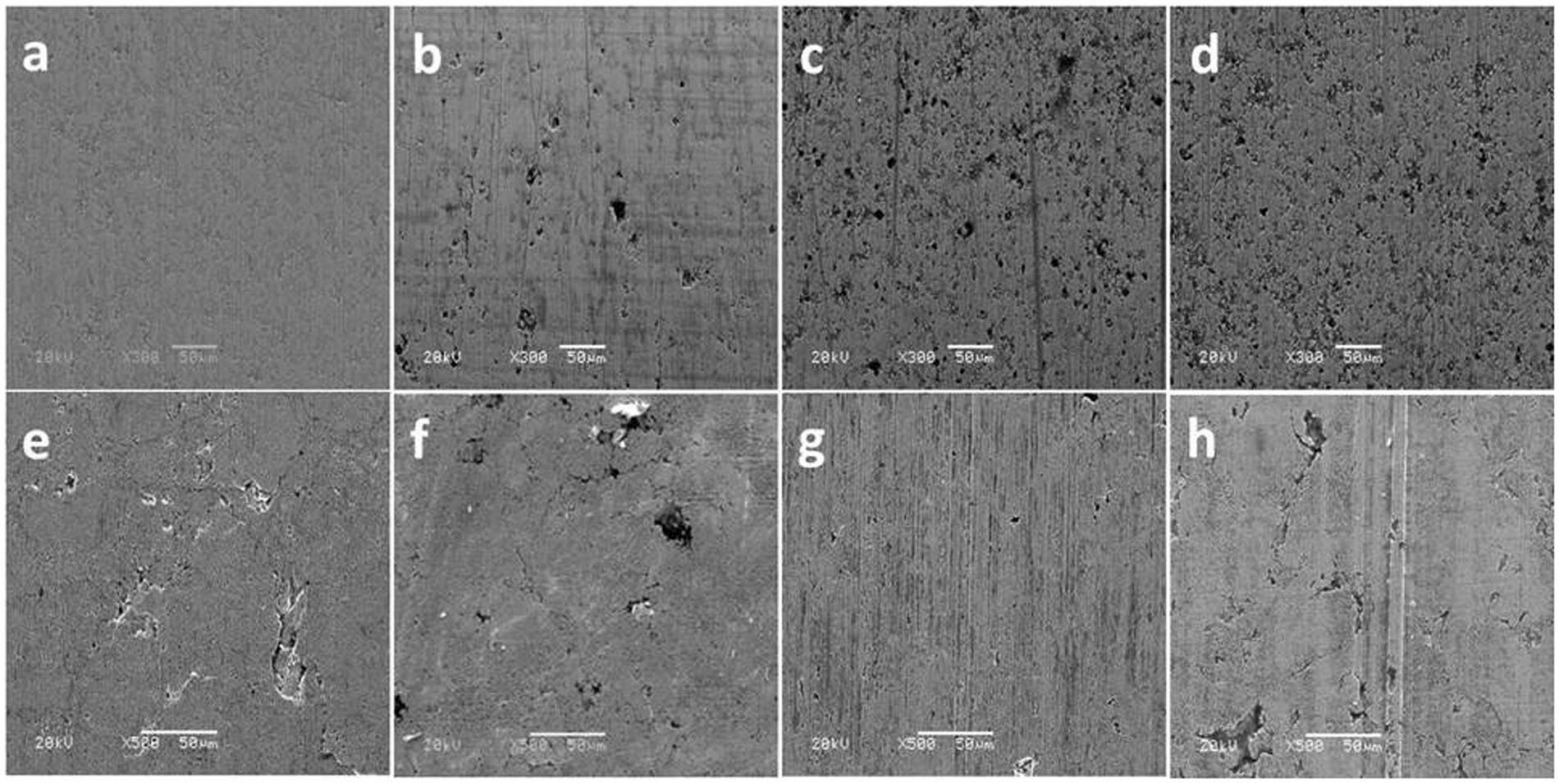
SEM images of the worn surfaces of CrN coated ring (**a**,**b**,**c** and **d**) and the opposing counterparts (**e**,**f**,**g** and **h**) in different sliding periods: (**a**,**e**) 1 h; (**b**,**f**) 2 h; (**c**,**g**) 4 h; (**d**,**h**) 8 h.

Contacting areas of the CrN/cast iron couples were observed by SEM, as shown in Fig. 3. As the sliding continued, the surface morphologies evolved. After 1–2 hours of sliding, the worn CrN surface was relatively smooth and featureless showing normal polishing wear, (Fig. 3a and b). With extended sliding pits and grooves appeared on the worn CrN surfaces (Fig. 3c and d). The dominant wear mode was abrasive wear. Figure 2 shows that the wear rate for the CrN ring is linear with respect to time. However, the cast iron counterpart liner shows an increasing rate of wear with time. Although some pits and spalling occurred in some local areas as well as micro-cracks, the worn cast iron surfaces were initially relatively smooth (Fig. 3e,f). As sliding proceeded, the reactive film derived from lubricant became thinner and eventually was unable to separate the metallic contacts effectively, and grooves appeared on the counterpart after 4 h sliding (Fig. 3g). After 8 hours of sliding, clear abrasion and severe deformation resulted in spalling and cracking (Fig. 3h).

**Figure 4:**
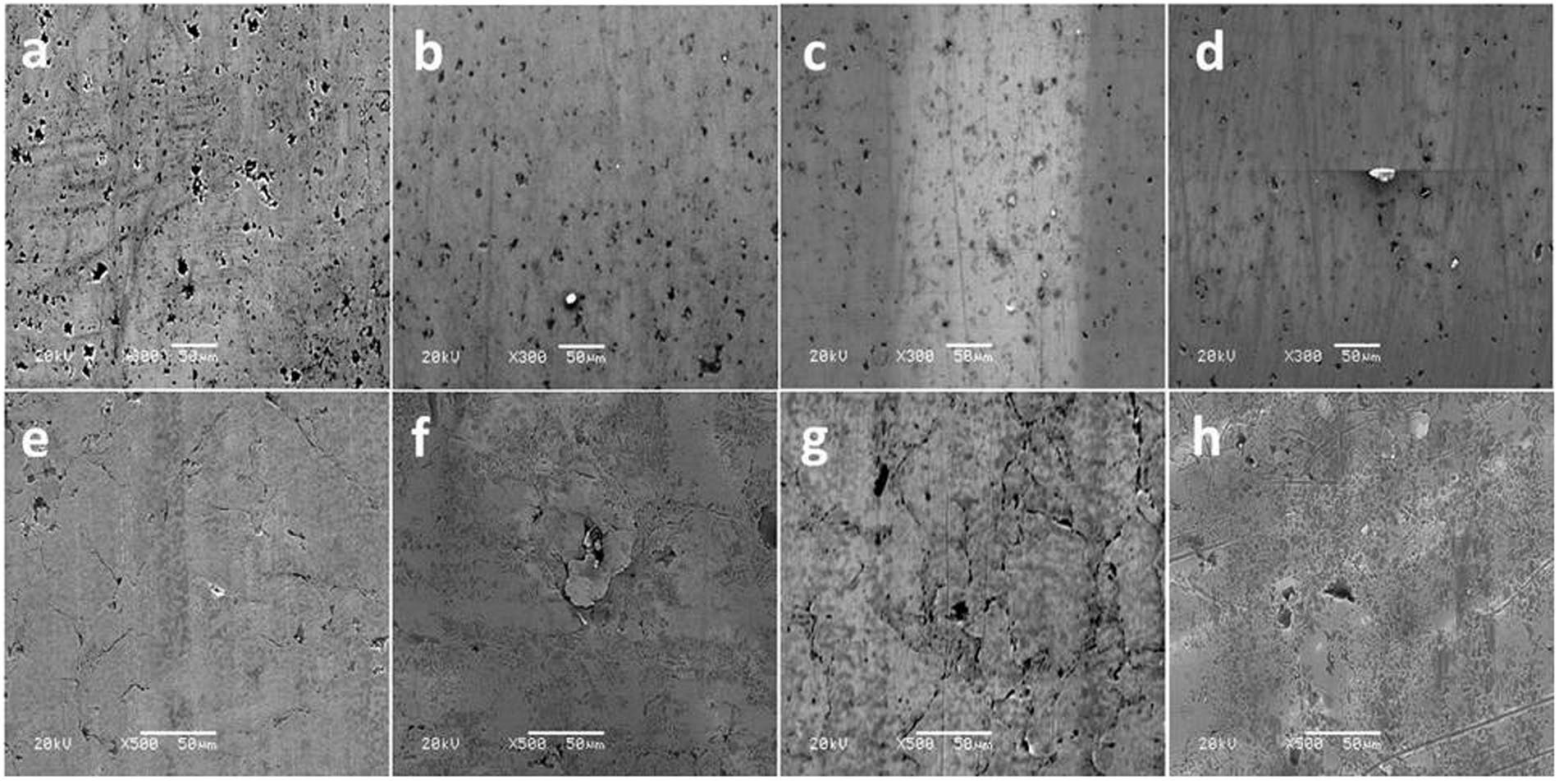
SEM images of the worn CrN + GLC ring surfaces (**a**,**b**,**c** and **d**) and the cast iron contacts (**e**,**f**,**g** and **h**) in oil lubrication: (**a**,**e**) 1 h; (**c**,**f**) 2 h; (**c**,**g**) 4 h; (**d**,**h**) 8 h.

The morphologies of the worn CrN + GLC couple were very smooth (Fig. 4). All the CrN + GLC surfaces showed typical polishing wear. In this study, the intermediate CrN layer was grown by arc ion plating deposition^18^. Surface defects in this layer, such as protrusions, were therefore present after coating with the GLC. The 1 hour sliding surface showed a number of micro-voids and pits as a result of the removal of such surface protrusions in Fig. 4a. After 2 hours sliding, some wear debris began to fill some of the pits (Fig. 4b), and this proceeded with further sliding, resulting in very smooth surfaces (Fig. 4c,d).

The opposing cast iron surface showed common wear characteristics in Fig. 4e,h, including spalling, cracks, and pits due to material being removed by polishing wear. At some locations there were some black tribofilm stripes on the worn surface. After 8 hours of sliding, the worn surface of the cast iron was more porous but the cracking has disappeared - possibly filled and closed due to the accumulation and compaction of wear debris (Fig. 4h). Previous research claimed that the porous structure on the cast iron surface was an inherent property of the cast iron material^19^.

In the case of CrN, under boundary lubrication, the resultant tribofilm inhibited the plastic deformation of asperities to some extent^20, 21^, but the valleys of the ploughing grooves and the surface microcracks became increasingly intensive on the worn liner surfaces as the duration extended, as shown in Fig. 3. This was associated with the gradual depletion of tribofilm as well as corrosion-fatigue cracking^22, 23^. In contrast, the application of a GLC layer effectively alleviated plastic deformation and abrasive wear on both counterparts (Fig. 4). Comparison of the morphological evolution in Figs 3 and 4 shows that the GLC surface was much smoother than that of the the CrN surface. This indicates that GLC provides enhanced wear resistance even in situations where lubrication is marginal.

### SEM/EDS Analysis on the worn surface

SEM/EDS confirmed lubricant-derived species were present on the worn CrN and corresponding cast iron surfaces. Clear signals of Zn, P, S, and O elements could be found as well as Cr and Fe. A significant fraction of Ca was also present, originating from the detergent in the oil formulation (calcium sulfonate) in Figures S1 and S2 (Supplemental information). The EDS analysis showed that the chemical interaction occurring between additives in the oil and solid CrN/cast iron contacts, formed an effective tribofilm that separated the contact from friction and wear^16^.

Some researchers claimed that a tribofilm forms on a DLC/steel pair under boundary lubrication^24^. When the worn GLC surface in this work was probed by EDS, none of the elements originated from the lubricant were detected (Figure S3). High levels of Zn, P and S were detected on the corresponding cast iron surfaces (Figure S4). During sliding, some of the carbon coating would have either detached or otherwise worn and would have ended up compacted into the tribofilm. A key question is how might such wear debris form a chemical tribofilm and influence the tribological performance. SEM-based EDS lacked the sensitivity to probe the existence of carbon species within the tribofilm. Raman and TEM characterization were therefore used.

### Raman analysis

**Figure 5:**
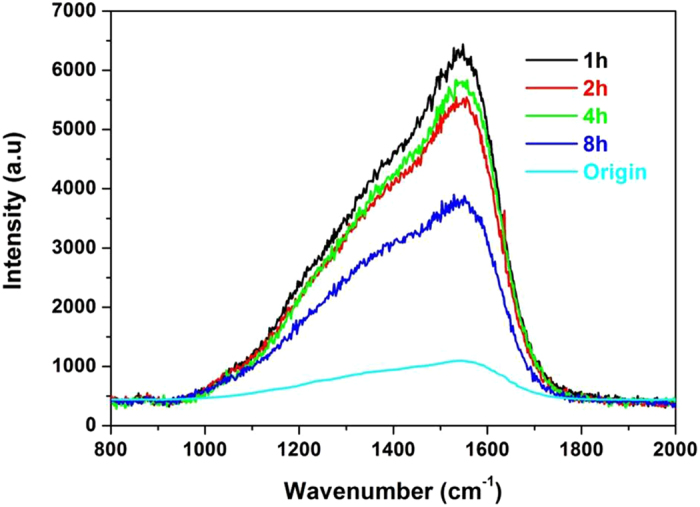
Raman spectra of worn GLC surfaces after different sliding durations.

Raman spectra from CrN + GLC/cast iron contacts enabled characterization of the various worn surfaces. The Raman spectra from GLC coated surfaces as a function of sliding duration are shown in Fig. 5. Raman spectra obtained from the worn GLC surfaces consisted of a relatively sharp peak at approximately 1560 cm^−1^ and a shoulder peak at about 1385 cm^−1^, which corresponded to the graphite in-plane mode (G-band) and the disorder mode (D-band), respectively^25^.However, no Raman peaks characteristics of metallic oxides were detected on the GLC surface, so the transfer of iron oxides from cast iron to GLC was insignificant. Multi-peak Gaussian fitting of Raman spectra was applied to illustrate the structural evolution of the GLC surfaces, and the results of this analysis are shown in Table 1. The original GLC surface showed a high I_D_/I_G_ ratio of 1.85, which meant more sp^2^-bonded carbon in an amorphous formation. As compared to the original GLC surface, the positions of the D and G peaks obtained from the worn GLC surfaces did not shift, but the I_D_/I_G_ ratios displayed a clear rise, which indicated more and more graphitic carbon occurring at the near-surface region.

**Table 1:** Fitted Raman spectra of different GLC surfaces: Position and FWHM of D and G peaks as well as the I_D_/I_G_ ratios.

Sample	D peak (cm^−1^)	FWHM of D peak (cm^−1^)	G peak (cm^−1^)	FWHM of G peak (cm^−1^)	I_D_/I_G_
Original GLC surface	1366.6	342.03	1565.32	181.7	1.86
CrN + GLC-1h	1380.61	367.57	1555.02	158.4	2.21
CrN + GLC-2h	1385.71	382.27	1558.01	160.53	2.60
CrN + GLC-4h	1389.34	347.51	1563.07	154.55	2.32
CrN + GLC-8h	1388.74	390.57	1558.16	152.53	3.53

The ratio of I_D_/I_G_ gave the information of structural disparity on the worn GLC contacting area. After 1 h, 2 h and 4 h sliding, the I_D_/I_G_ ratios were 2.21, 2.60, and 2.32, respectively. This showed that the worn GLC surface maintained the similar structure, and that the boundary tribofilm was still effective. Since the tribochemical layer is thick enough to accommodate wear particles such as carbon debris, this tribofilm acted as an inhibitor which slowed down graphitization of GLC surface^25^. However, after 8 hours of sliding a marked increase in the I_D_/I_G_ value to 3.53 indicated that severe graphitization had occurred. It is hypothesized that the tribofilm became too thin to avoid bulk contact, the combination of high shear stress and temperature would be directly applied onto the contacting surface. This accelerated the phase transformation from amorphous into oriented graphite-like species.

**Figure 6:**
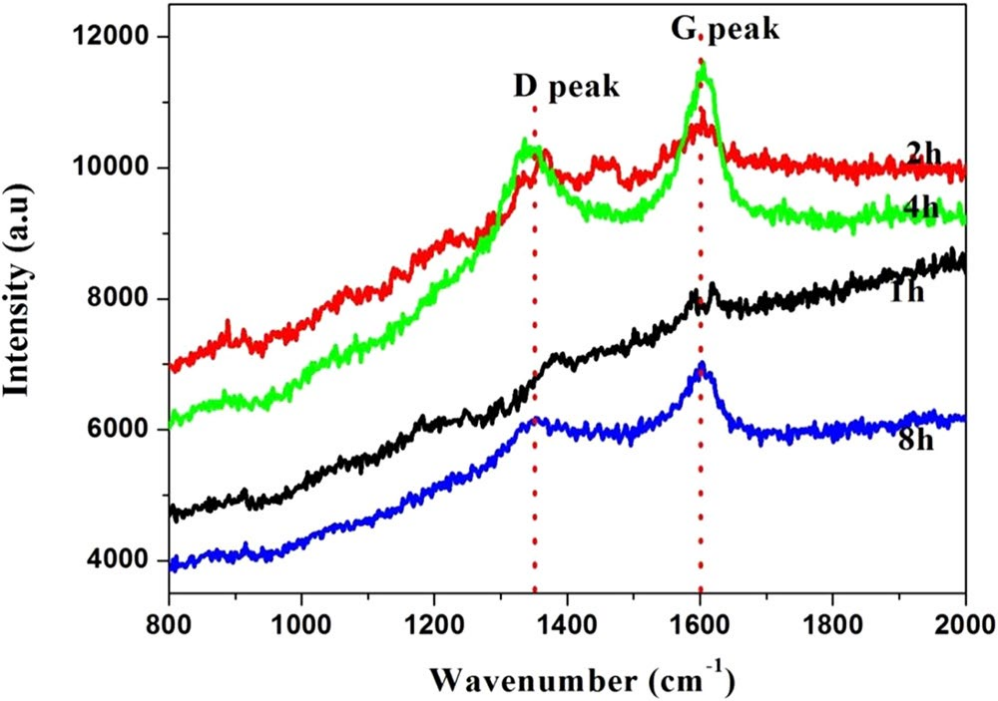
Raman spectra of the cast iron surfaces sliding against GLC after different durations.

It has been argued that it is difficult to transfer carbon onto the counter surface under boundary lubrication^26, 27^. Raman analysis of the cast iron surfaces are shown in Fig. 6. The typical characteristics of D and G peaks within these spectra confirm that graphitic carbon is present on the cast iron surfaces, and the structure is quite different to that of the worn GLC surface (Fig. 5). All spectra showed the D peak clearly resolved from the G peak. These peaks broadened and intensified as sliding continued. The Gaussian deconvolution was also applied to fit these Raman spectra, and the results were summarized in Table 2. The sharp G peaks and the FWHM values indicate that the deposit on the cast iron was predominantly sp^2^-bonded carbon with a degree of disorder. The Raman spectra clearly demonstrate that structural ordering of the carbon occurred at the near-surface region of GLC even after a shorter sliding. During sliding, carbon was transferred from the GLC onto the cast iron counter surface forming an ordered deposit.

**Table 2:** Fitted Raman spectra of the worn cast iron surfaces: Position and FWHM of D and G peaks.

Sample	D peak (cm^−1^)	FWHM of D peak (cm^−1^)	G peak (cm^−1^)	FWHM of G peak (cm^−1^)
CrN + GLC-1h	1388.52	142.87	1598.72	135.16
CrN + GLC-2h	1360.31	84.59	1600.23	138.83
CrN + GLC-4h	1341.17	131.97	1604.87	73.87
CrN + GLC-8h	1356.13	169.32	1598.20	87.52

The exact structure and disposition of this deposit was of considerable interest., since it could provide insights into how the carbon improves wear resistance by eliminating three-body wear between the rubbing contacts. There was also the question as to origin of the solid carbon at the interface, since it could have derived from decomposition of the oil or from graphite in the cast iron itself. Raman analysis was applied to the CrN/cast iron contact (Figure S5). Although graphite flakes embedded in the cast iron matrix were detected (Figure S6), the spectra from the sliding contact of CrN/cast iron couple did not show carbon peaks. This shows that the carbon found on the cast iron counterface of the GLC/cast iron couple was a direct result of transfer from the GLC and did not arise from the cast iron or from oil decomposition. To better understand the disposition and nature of the carbon in the tribofilm, TEM/EELS characterization was carried out.

### TEM analysis

**Figure 7:**
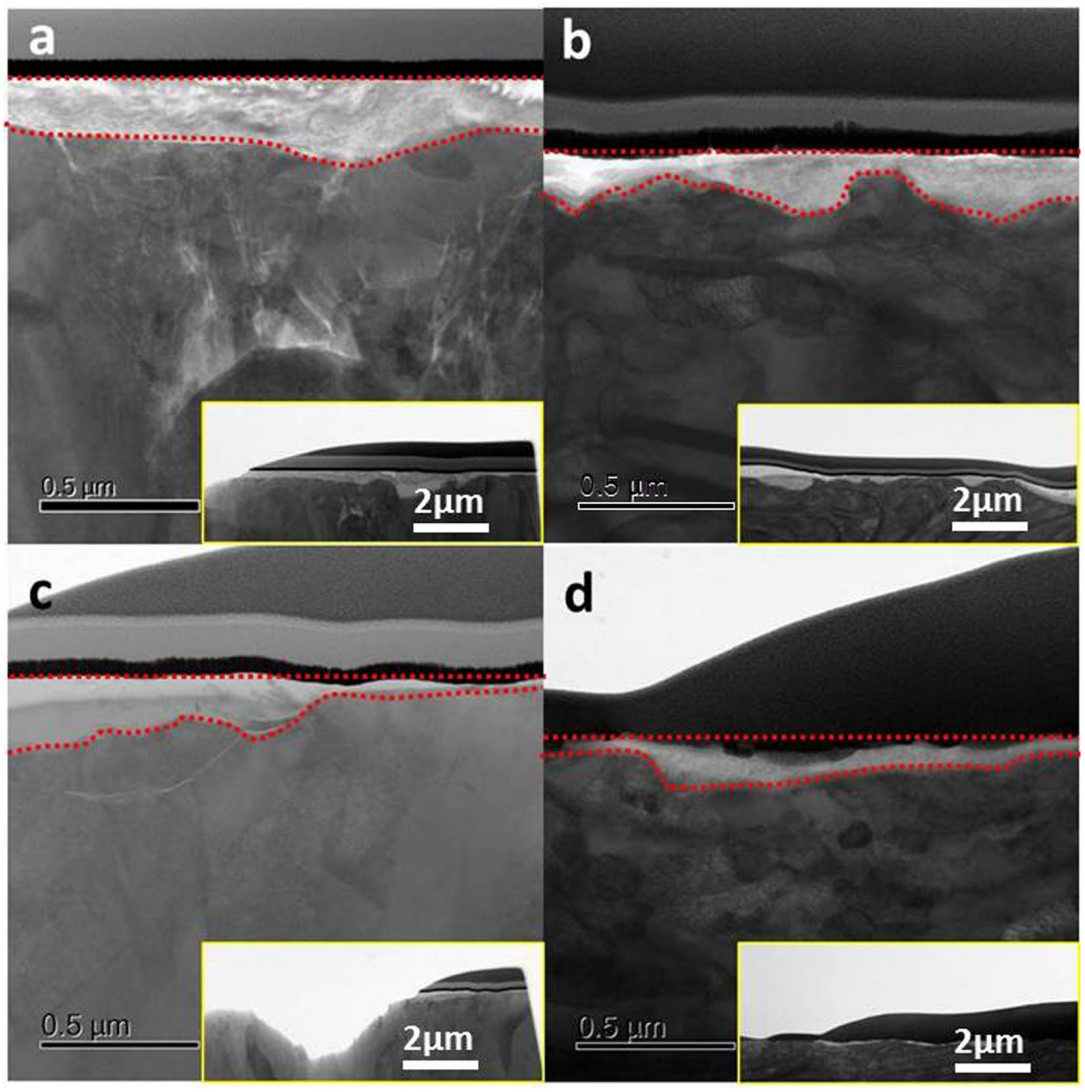
Tribofilm obtained from the CrN/cast iron coupled interface after 1-hour (**a,b**) and 8-hour (**c,d**) rubbing: (**a,c**) CrN surface, (**b,d**) cast iron liner.

**Figure 8:**
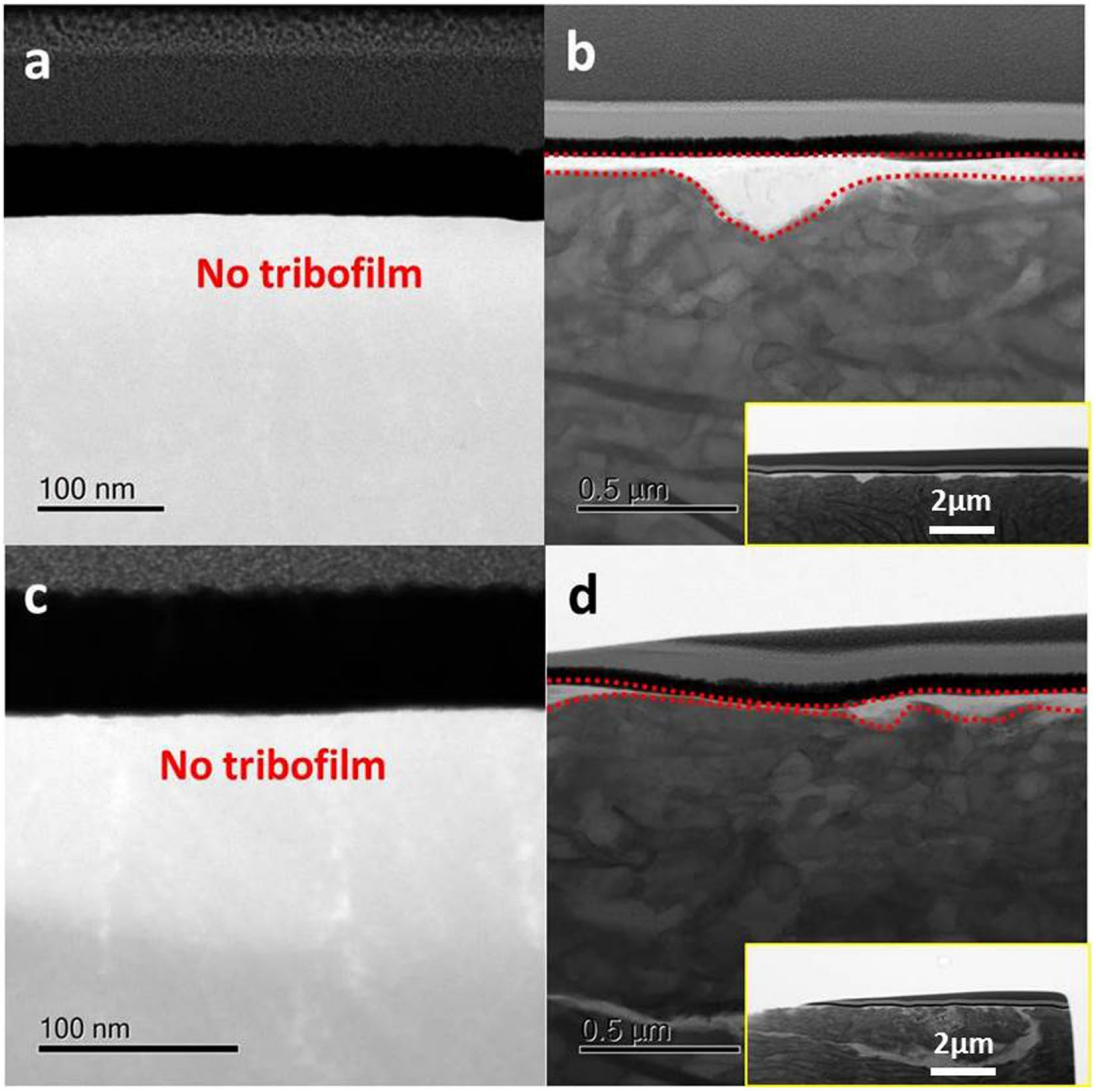
Tribofilm obtained from the GLC + CrN/cast iron coupled interface after 1-hour (**a,b**) and 8-hour (**c,d**) rubbing: (**a,c**) CrN + GLC surface, (**b,d**) cast iron liner.

Cross-sectional TEM images of the tribofilm on the cast iron sliding against CrN and GLC are shown in Figs 7 and 8. For the tribofilm at the CrN/cast iron interface in Fig. 7, the CrN surface was covered by a relatively thick ZDDP-derived tribofilm (around 200 nm thick) after 1-hour of sliding (Fig. 7a), while the corresponding cast iron surface produced a ca 160nm-thick polyphosphate nanocomposite film in Fig. 7b (the tribofilm is outlines in red in Fig. 7). After 8 hours rubbing the tribofilm thickness on the cast iron and corresponding CrN surface both decreased to ~60 nm (Fig. 7c and d). The observations were consistent with previous investigations^8, 13, 14^.

The tribofilm formed on the CrN + GLC /cast iron contact were quite different (Fig. 8). A distinct tribofilm formed on the cast iron surface (Fig. 8b and d) but was absent from the CrN + GLC surface (Fig. 8a and c). After sliding for 1 hour, the cast iron surface yielded a ~130nm-thick tribofilm, which reduced to no more than 40 nm thick after 8-hours of sliding. The results were consistent with the experimental parameters. In this study a limited quantity of oil was supplied during sliding and thus lubrication diminished with increasing sliding time. This caused the tribofilm to become thinner with time. This result highlights the sacrificial nature of the boundary tribofilm derived from oil additives^8, 13, 14^.

It was further noted that, the cast iron sliding against CrN exhibited a higher degree of surface roughness than cast iron slid against CrN + GLC, especially at the initial 1-hour running-in period. This is most apparent in the insets in Figs 7b and 8b. The CrN + GLC surface was very effective at polishing the mated cast iron surface. With regard to the uniformity of tribofilm, tribofilms on cast iron against CrN + GLC were relatively uniform. However, in the case of the GLC surface no tribofilm formed (Fig. 8a and c). This was consistent with the Raman result which showed no metal oxide transfer to the GLC surface (Fig. 5). For the CrN/cast iron contacts, the tribochemical film readily developed on the CrN surface during sliding (Fig. 7a and c).

### TEM/EDS Analysis from the cross-sectional view

In the case of ZDDP-derived tribofilms on ferrous surfaces, these normally appear with a compositional gradient across the film thickness^28^. Some interesting characteristics of the elemental distribution across the tribofilm thickness were found by combined STEM/EDS in Figures S7–S10. (i) The distributions of Zn and S were closely correlated, which was consistent with the published work by Qu *et al*.^29^. If the case of cast iron slid against CrN, high concentrations of Zn and S were located at the outer surface of the tribofilm after 1-hour sliding and at the bottom of the tribofilm after 8-hours of sliding, as shown in Figures S7d and S8d. In the case of cast iron slid against GLC, the Zn and S were mainly present at the outer surface of tribofilm on cast iron (Figures S9d and S10d). (ii) The distributions of Ca and O were closely correlated through the thickness, whilst the peaks of Zn and S emerged at the bottom of tribofilm. (iii) The tribofilm elemental distributions on the CrN surface (Figs S7b and 8b) were somewhat different to those on the corresponding cast iron surfaces (Figures S9d and S10d). For example, the peak of Zn and S was found near the centre of tribofilm. (iv) no tribofilm formed on the CrN + GLC surface in Figures S9a and S10a. Although small amounts of S, P and Ca, etc. from the lubricant were detected at the surface (Figures S9b and S10b), no compositionally discrete layer was located.

### TEM/EDS-based phase mapping

ZDDP-derived tribofilms have a heterogeneous composition with a hierarchical structure across the thickness^29^. In this work, the applied lubricant was a commercially formulated oil. The resultant tribofilm consisted of mainly zinc-iron phosphates and sulfide (or sulfate) with a certain amount of CaCO_3_^30^. However, the exact constituents of the compound phases and their spatial distribution across the tribofilm has been poorly studied to date.

According to the hard and soft acids and bases (HSAB) principle^31^, hard base phosphate reacts preferrentially with hard acids such as Fe^3+^ ^32^,. Soft bases, such as S^2-^, interact strongly with soft acids, such as Zn^2+^ in preference to harder acids such as Fe^2+^ or Fe^3+^ ^32, 33^. For Zn^2+^, ~22% of the Zn has been found to be associated with zinc phosphate and unreacted ZDDP^32^. As for the S element, the amount of hard base SO_4_^2−^ primarily appeared at the super facial of sulphide compounds^34, 35^. It has been claimed that calcium carbonate (CaCO_3_) exists within the tribofilm, but it was very difficult to differentiate between calcium carbonate and calcium phosphate. Carbonate in the form of CaCO_3_ has been found to dominate during the early stages of sliding^36^. Since Ca^2+^ is a harder acid than Zn^2+^, there is a strong affinity for calcium phosphate to form in preference to zinc phosphate. Oxygen in the tribofilm was mostly associated with phosphate, carbonate and sulfate, along with metallic oxides such as Fe_2_O_3_ or CaO. Figures S7–S10 showed that tribofilm mainly consisted of Fe, Zn, Ca, S, P with a large amount of O. The compounds within tribofilm are very complex, and so phase mapping was used to understand the chemistry, spatial distribution and retention of various compounds as a function of sliding couple and duration. The possible compounds within the tribofilm in this study were therefore simplified and hypothesized to be FePO_4_, Zn_3_(PO_4_)_2_, Ca_3_(PO_4_)_2_, ZnS, FeS, CaCO_3_, CaSO_4_, along with iron oxides. Individual compounds were identified using the built-in phase mapping tools (Compass) available within the Noran System Seven software (Version 3.3, https://www.thermofisher.com/au/en/home/industrial/spectroscopy-elemental-isotope-analysis/microanalysis-electron-microscopy.html).

**Figure 9:**
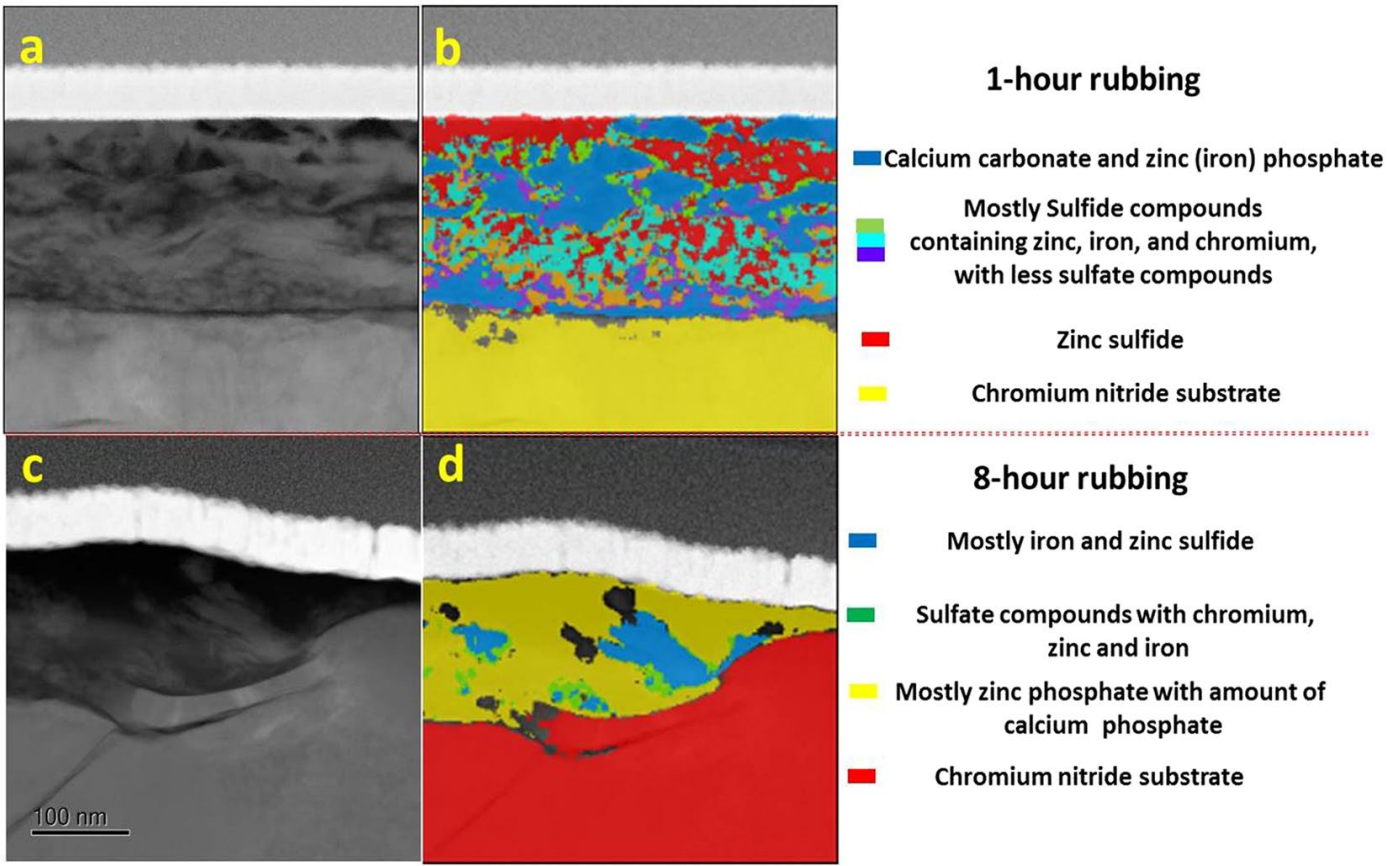
Cross-section TEM images and corresponding phase contrast images of tribofilm on CrN surfaces after 1-hour (**a**,**b**) and 8-hour rubbing (**c**,**d**).

Figure 9 showed the inhomogeneous distribution of compounds within tribofilms on the worn CrN surface. The phosphate and sulfide compounds appeared in higher concentrations at outer surface of the tribofilm in Fig. 9b and d. After the initial 1-hour sliding period, a high concentration of sulfide was found in the near-surface and intermediate regions in Figure S7b. After 8 hours of sliding the tribofilm consisted of mainly zinc phosphate with some calcium phosphate, within which a fraction of the sulfide compounds was dispersed (Fig. 9d).

**Figure 10:**
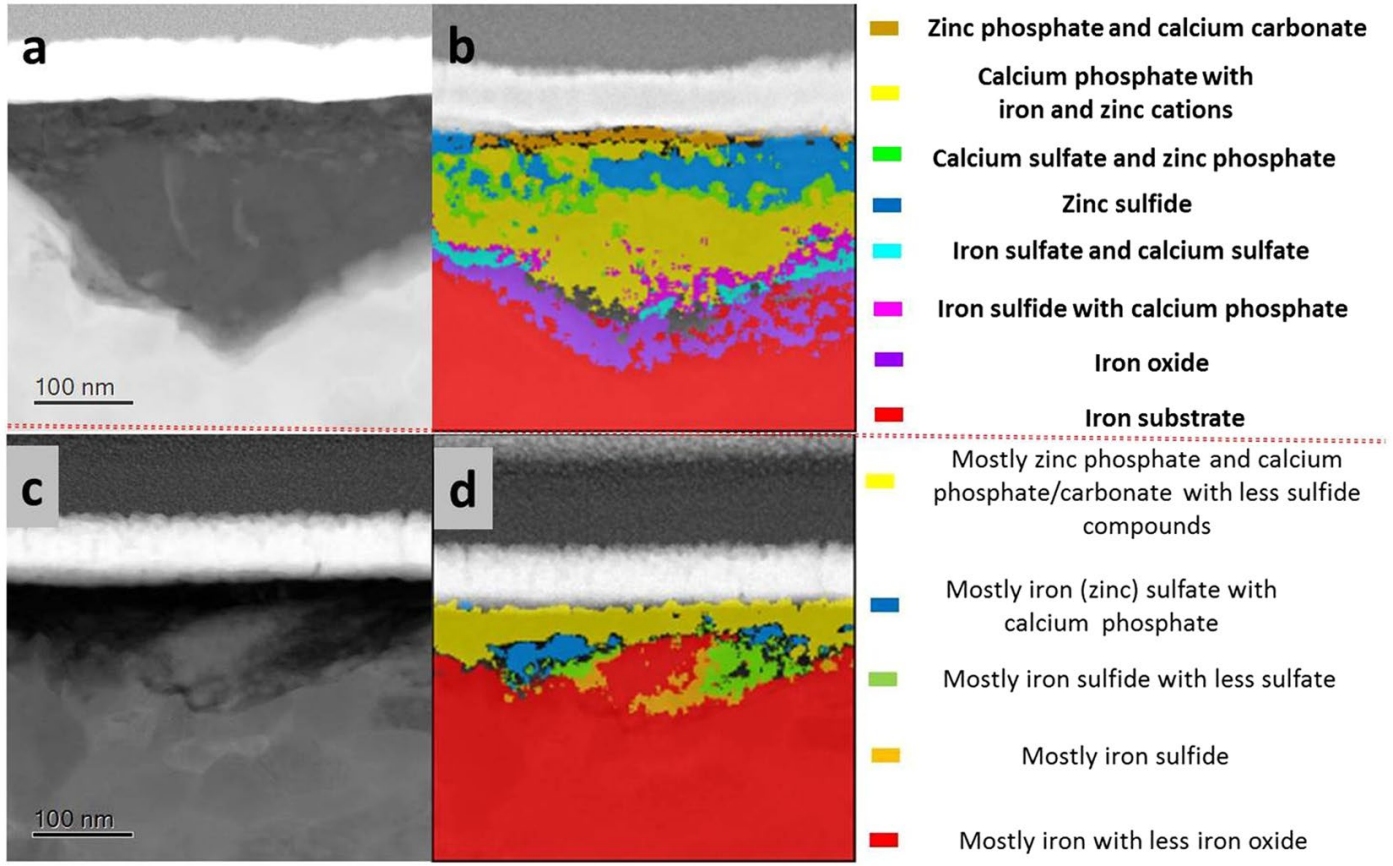
Cross-section TEM images and corresponding phase contrast images of tribofilm on cast iron rubbing against CrN surface after 1-hour (**a**,**c**) and 8-hour rubbing (**b**,**d**).

Figure 10 showed the phase maps of tribofilms formed on cast iron after sliding against CrN. Different phases occurred in different locations, and these varied with sliding time (Fig. 10b and d). After 1 hour of sliding the tribofilm (Fig. 10b) showed that zinc sulfide was mostly located in the near-surface region, while a mixture of zinc phosphate, calcium carbonate and sulfate was present within the middle region, and a thin film of iron sulfide was lying on the transitional iron oxide near the metal interface. After 8 hours of sliding (Fig. 10d), the near-surface region of tribofilm were mostly zinc phosphate and calcium carbonate/phosphate, and the iron (zinc) sulfides were near the metal interface.

**Figure 11:**
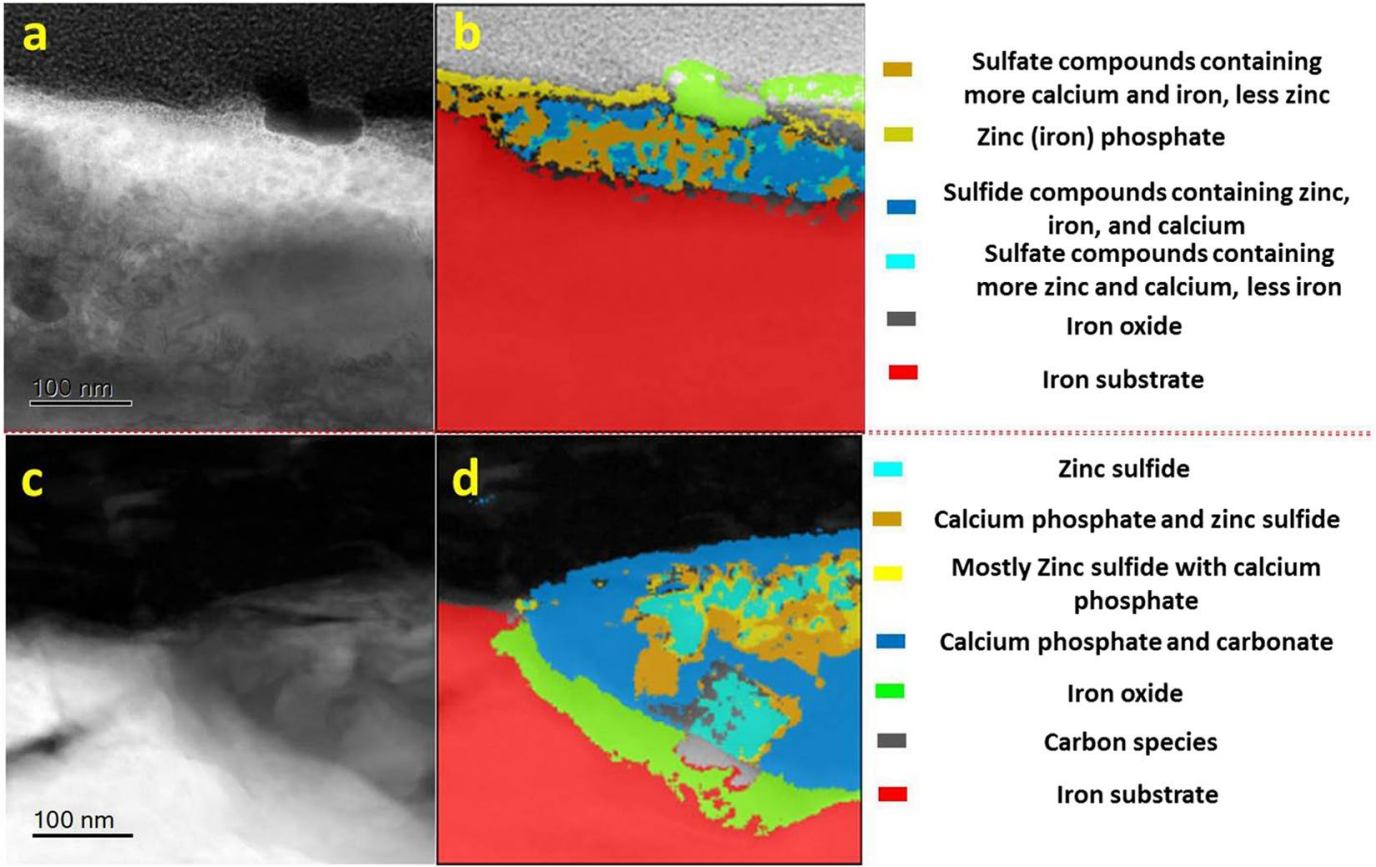
Cross-section TEM images and corresponding phase contrast images of tribofilm on cast iron rubbing against CrN + GLC surface after 1-hour (**a**,**c**) and 8-hour rubbing (**b**,**d**).

Since there was no tribofilm on the GLC surfaces (Figures S9 and S10), only the tribofilms on the opposing cast iron surfaces are shown in Fig. 11. Figure 11b showed zinc phosphate with an amount of calcium carbonate at the near-surface after 1 h sliding. Sulfide and sulphate compounds comprised the bulk of the film. After 8 hours of sliding, the tribofilm in Fig. 11d primarily contained calcium phosphate and carbonate with amount of zinc sulfide concentrated at the middle region.

Raman analysis indicated the presence of graphitic carbon in this material, probably present in the form of fine particles or as a film formed within or over the polyphosphate tribofilm in Fig. 6. A thin carbon film was found to be deposited in Fig. 11c, and it differed from the protective carbon before the FIB operation. Especially, an amount of calcium carbonate was even figured out within such carbon deposition in Fig. 11d. This strongly indicated that a graphitic carbon lying over the reactive tribofilm.

Another interesting aspect was the presence of calcium-related compounds throughout the tribofilm regardless of the sliding duration. In this study, they were derived from the detergent additive in the oil which clearly influenced the formation of the tribofilm. Since calcium was a strong base, it would neutralize all kinds of acidic products such as carbonate, phosphate, and sulfate, as well as organic acids, as shown in Figs 9–11. On the other hand, these active bivalent Ca ions could promote the local aggregation and retention of other compounds like phosphate and sulfide inclusions. Since the retention of these inclusions was not driven by the surface chemistry, it must be associated with local physical and chemical inhomogeneties such as small chemical heterogeneities^28, 37^.

### TEM/Diffraction characterization

**Figure 12:**
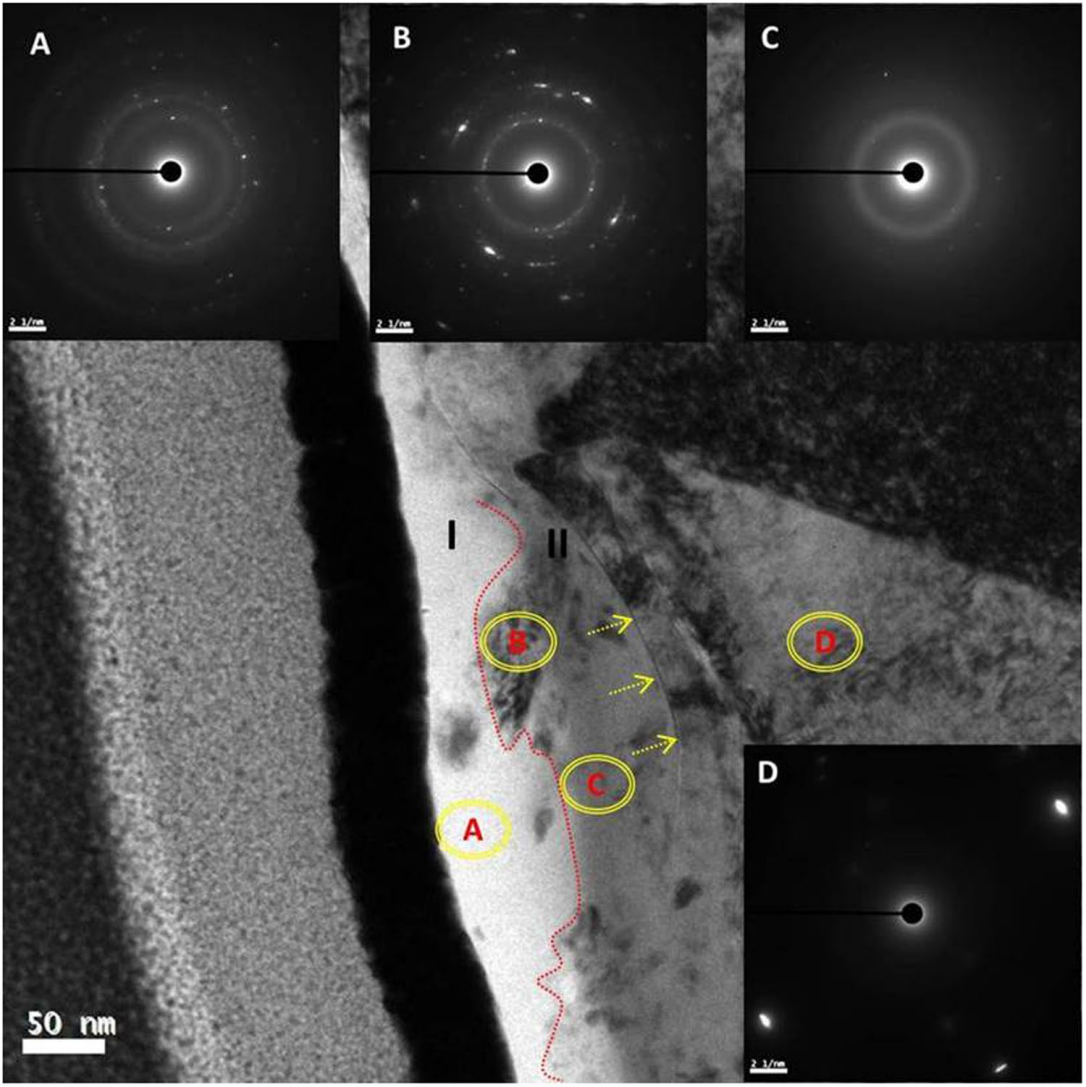
Bright field TEM image in cross-secitonal view and selected area electron diffraction (SAED) patterns (**a,b,c** and **d**) across tribofilm on the worn CrN surface after 1-hour rubbing.

The bright field HRTEM image in Fig. 12 demonstrated the tribofilm on CrN consisting of two distinct layers. The bright layer in region I was 10~30 nm thick, while the relatively grey layer in region II was ~50 nm thick. Accordingly, the region labelled A in the polyphosphate tribofilm in region I was confirmed as being amorphous by the SAED pattern (Fig. 12A) and Fig. 12C. Note the weak, sharp crystalline reflections in this pattern originate from the adjacent highly crystalline protective Au layer added during FIB preparation. The B and C area in region II clearly displayed the diffraction spacings, arising from iron oxide phases. The SAED pattern (Fig. 12B) in the B area had lattice spacings which corresponded to (110) and (113) of hematite Fe_2_O_3_, accompanied by another spacing arising from (220) of magnetite (Fe_3_O_4_). The SAED pattern in region D showed strong (200) lattice spacings of FCC CrN (Fig. 12D). The TEM/diffraction results well demonstrated iron oxide particles were either at the surface/layer boundary or independently suspended within the tribofilm. In contrast with the tribofilm interface at case iron, a distinct interfacial boundary between the tribofilm and CrN interface (arrowed in Fig. 12) indicated an absence of oxide layer on the CrN substrate.

**Figure 13:**
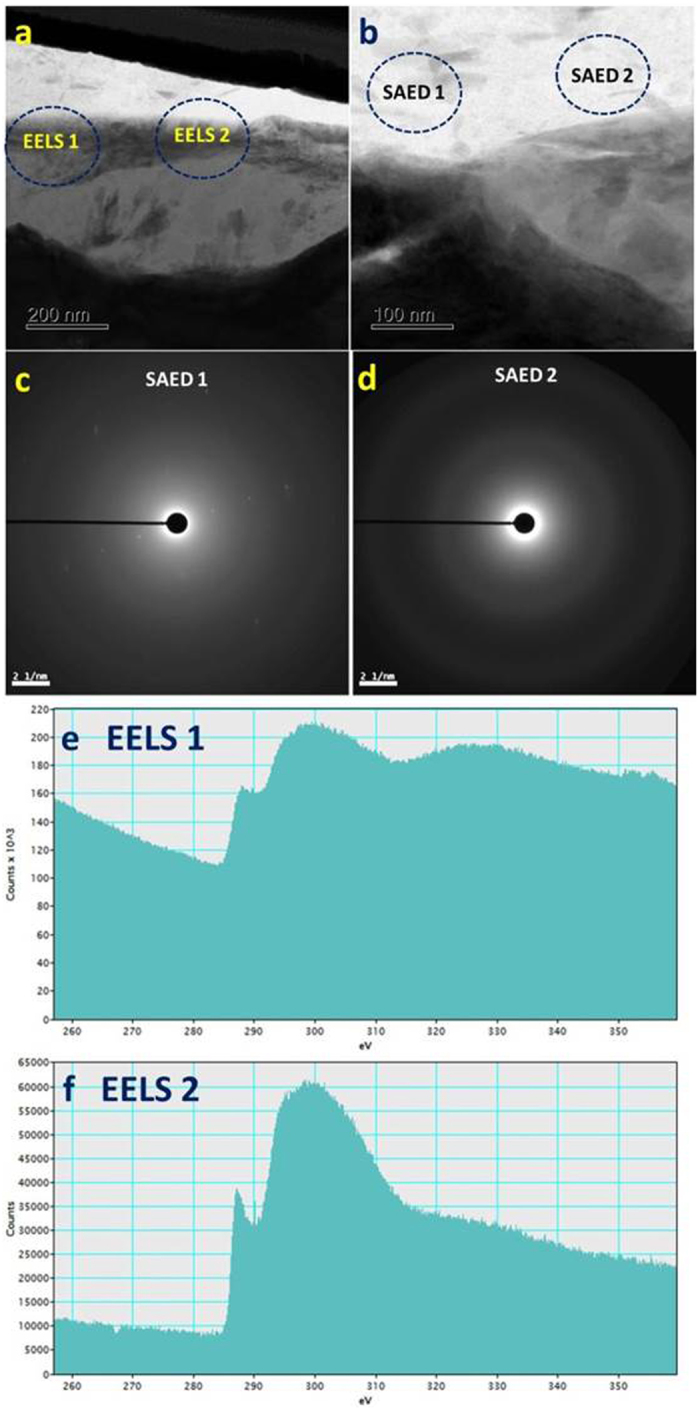
Bright field TEM images and associated SAED patterns and EELS spectra confirming the existence of a carbon-rich layer on the the tribofilm formed on cast iron sliding against GLC for 8 h: (**a**) Bright field TEM image showing regions analysed with EELS; (**b**) Bright field TEM image showing regions analysed with SAED; (**c**) SAED pattern from region SAED1 in (**b**); (**d**) SAED pattern from region SAED2 in (**b**); (**e**) Carbon K EELS spectrum from region EELS1 in (**a**); (**f**) Carbon K EELS spectrum from region EELS2 in (**a**).

Raman analysis confirmed the presence of transferred carbon on the cast iron counterpart (Fig. 5). However, due to low sensitivity for mapping carbon, mapping did not locate a discrete carbon-rich layer (Fig. 9); an oriented graphitic carbon film would be expected to form over the tribochemical film if the lubricating conditions became increasingly oil-starved. Electron energy loss spectroscopy (EELS) (combined with SAED analysis) was able to identify a carbon-rich layer in the tribofilm (Fig. 13a and b). The SAED patterns show diffuse rings which show the superficial carbon film is amorphous (Fig. 13c and d), where carbon crystallite of less than 1 nm was included in accordance with the Raman analysis shown in Fig. 6.

The EELS spectra had two main features: a shoulder peak at ~285 eV and a broad band with a maximum at ~300 eV, which corresponded to 1s-π*and 1s-σ* electronic transitions, respectively. The former was associated with sp^2^-bonded carbon atoms and the latter with both sp^2^ and sp^3^ carbon atoms^38^. The EELS analysis unambiguously identified the layer as being very carbon-rich, and the fine structure of the C K EELS edge well indicated the amorphous nature of carbon, which was consistent with the Raman characterization (Fig. 6). Since the tribofilm was not able to protect the moving contact sufficiently as the lubrication deteriorates, more carbon debris were transferred onto the opposing surface and restored with a layer enriched with graphitic composition (Fig. 6). This carbon layer over the reactive tribofilm adapted the graphitization occurring on the opposing GLC surface, delivering the steady friction of the CrN + GLC/cast iron pair in Fig. 1b. However, if the materials loss was defined by the specific wear rate, the loss rate of material accelerated on the GLC surface. For example, the specific wear rate was 2.89 × 10^−9^ mm^3^/N·m after 4-hour and 3.85 × 10^−9^ mm^3^/N·m after 8-hour of rubbing, respectively. This result indicated the transferred carbon stabilizes the friction at the expense of GLC itself loss.

## Discussion

Many engine components experience boundary lubrication, where a thin solid film exists as a result of the complex thermo-mechanical interaction of lubricant/contacting surfaces. The resulting friction and wear characteristics are governed by this tribofilm. The interaction between lubricant additives and such surfaces is the key focus in this work. The underpinning mechanisms of these interactions were evaluated under the increasingly oil-starved conditions used in this study. The results demonstrated the composition of tribofilm from the commercial oil was mainly zinc-iron polyphosphate and sulfide as well as calcium-related detergents from the characterizations of SEM/EDS, TEM/SAED/EDS and Raman analysis in this work.

### Lubricating and Wear Mechanism at CrN/cast iron surfaces

For the ferrous contacts, a 50~150 nm tribofilm was reported to be always situated above solid surfaces under boundary lubrication^13, 16^. The thickness and compositional ratios within such a film vary with the conditions (e.g. pressure, velocity and temperature). The composition of this film, which can include Zn, Fe, S, P and O is laterally and vertically heterogeneous; a friction-induced stratified structure with a hierarchical composition develops across the tribofilm thickness^17, 18, 30, 34^. Sulfides have been reported^17, 18, 30, 34^. They were be located between the polyphosphate tribofilm and ferrous oxide, and appeared as ‘patchy islands’. They were not continuous on the iron oxide. Aktary *et al*.^39^ observed the dynamic evolution of the pad structure by AFM and noted how the rubbing surfa. These gradually expanded into an almost continuous, but still pad-like structure separated by deep valleys.

Original CrN on the piston ring was relatively rough with the locations of Cr-enriched droplets and craters. The worn surface was polished during friction (Fig. 3a and b). The tribofilm derived from ZDDP is compatible with nitride surfaces, and eventually a continuous protective film formed at the moving interfaces, (Fig. 7a and c); these results concur with Aktary’s and Qu’ s observations^10, 39^. The friction remained constant due to the presence of tribofilm in this study, but the CrN/cast iron contacts showed a gradual increase in wear with sliding time (Fig. 2). Although a tribofilm existed at the CrN/cast iron interface (Fig. 7c and Figure S8), the tribofilm became progressively thinner as the sliding continued without oil replenishment (Fig. 7), which finally resulted in an increased wear rate.

Since additives which contain sulfur and phosphorus are chemically reactive towards ferrous-based surfaces, this facilitated formation of tribochemical film under boundary lubrication. In this study, the emergence of CrN surface would change the composition and structure of tribofilm, which was heavily dependent on the reactivity of additives with surfaces and their contacting properties. Pereira *et al*.^30, 34^ investigated the adhesive strength of tribofilms on a CrN surface. They found that the reactive tribofilm did not adhered to the CrN surface as strongly as on ferrous surfaces. Since CrN was thermally stable and chemically inert, it could not provide an oxide layer on the rubbing surface, so the organophosphate film was not able to bind to the surface via oxide as it did on the ferrous surface. In this work TEM investigation demonstrated that no transition region formed at the tribofilm/CrN interface (Figs 7 and 12). The adhesive strength of the tribofilm to CrN will be the subject of future studies.

**Figure 14:**
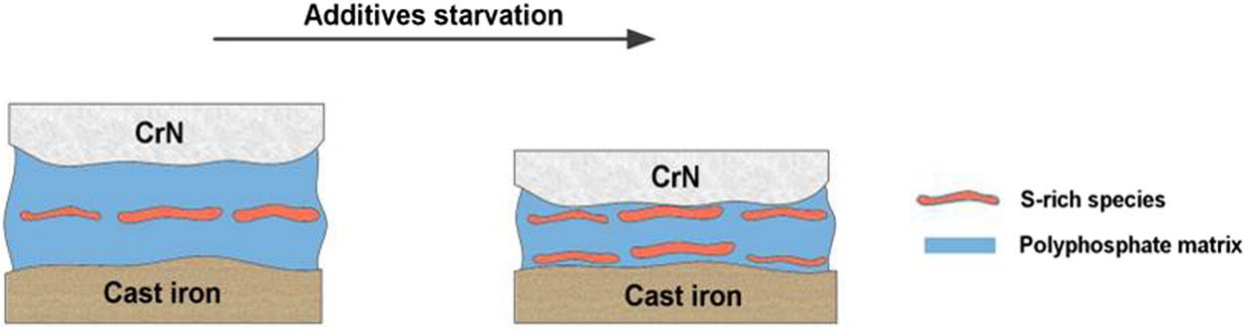
Schematic diagram showing the evolution of the tribofilm formed on CrN/Cast iron sliding couples as a result of lubricant starvation.

Phase mapping (Fig. 9a and b) showed that zinc sulfide was typically present in the near-surface region of the tribofilm on the worn CrN surface after 1-hour of sliding. A similar observation was made for films formed on cast iron surfaces (Fig. 10a and b). Podgornik *et al*.^9, 11, 35^ also found that sulfur in additives reacted with the CrN surfaces at higher contacting pressures and high addititive concentration to form chromium sulfide compounds. In this work extended sliding to 8 hours resulted in phosphate compounds dominating the tribofilm (Fig. 9c and d). Phosphates were located at the near-surface region while sulfide compounds were near the metal interface (Fig. 10c and d). It is known that the reduction in friction resulting from lubricant additives interacting with the surface is the result of reduced shearing strength within the tribofilm^36^. In this case, increasing friction at the cast iron/CrN contacts occurred with the increasing sliding periods. The concentration of sulfide compounds substantially reduced due to the diminishing lubrication. The results indicated that, sulfide compounds made significant contribution to friction reduction, while phosphate compounds were restored to combat wear. A schematic representation of the tribofilm evolution in CrN/cast iron contacts during lubricant starvation was shown in Fig. 14.

### Lubrication and Wear Mechanism at CrN+GLC/cast iron contacts

Components with amorphous carbon surface involves different acting mechanisms during friction, the graphitization of carbon itself, the formation of a chemically reactive layer, and the build-up of a transfer film on the opposite surface have always been considered^40^. However, the interactions between this carbon layer and lubricant additives, and the detailed tribological interactions are not well understood.

The unique friction behavior of amorphous carbon in dry sliding has been explained as the result of the transfer of sp^2^-carbon onto the counterpart^40^. When graphite-like amorphous carbon (GLC) was subjected to oil lubrication, it could produce energetic dangling covalent bonding during operation that can be passivated by adsorbed species such oil additives, which lowers the friction^40^; GLC is one type of amorphous carbon with higher fraction of sp^2^-carbon, and this was the reason for selecting it in this study. Some researchers claimed that lubricant would suppress the formation of a graphitic surface at the near-surface region of amorphous carbon and the transfer of carbon onto the opposite pair^27^. We observed an increasing graphitization of GLC as compared to original GLC surface in Fig. 5, while more graphitic carbon presented onto the opposite surfaces (Fig. 6). Raman analysis showed a graphitization of GLC was suppressed during 4 hour sliding, but after 8 hours of rubbing there was a sharp rise in the I_D_/I_G_ value. This difference was attributed to a competition between the formation of a chemical reactive layer and the build-up of a transferring carbon film over the tribofilm on the opposite surface, which basically depended on the concentration of additive. As we could not observe any carbon species in the tribofilm from the CrN/cast iron couples, the formation of carbon from oil decomposition was therefore excluded.

When sufficient additive is present, the graphitization of GLC was inhibited, while the carbon fine particles from GLC were believed to be dispersed into an amorphous phosphate matrix during stressed shearing where they provided the spherical rolling effect on reducing friction and wear^41, 42^. However, we could not find such a carbon distribution within the tribofilm due to the FIB foils being very thick, relative to the likely particle size. Raman spectra clearly identified graphitic carbon existing within the polyphosphate matrix in Fig. 6. TEM/EDS also showed the transfer carbon within the tribofilm onto the opposing surfaces. EDS lacks the analytical sensitivity for light element detection (in a higher atomic number matrix). Imaging, such as Fig. 11, showed some light-colored regions (low density) which were distributed randomly within the tribofilms, which may have been carbon-rich regions. A similar observation is reported in ref. 10. TEM also showed that the near-surface, carbon-rich region of tribofilm was perfectly amorphous^43^. The friction and wear results showed that, the generation of carbon nanoparticles did not degrade the anti-wear property of the reactive tribofilm, low friction without compromising the wear performance is mainly dominated by the reactive film in this work.

In this work, there is no physically adsorbed film on the worn GLC surface. Since the GLC specimen had been ultrasonically cleaned before the FIB sampling, any oil residue was believed to be removed. The following discussion focuses on the tribofilm on the opposing cast iron surface. As the concentration of additive gradually decreased with sliding time, the tribofilm on the cast iron became thinner and could not protect the contacting area completely (Figs 8 and S8). So as to adapt to an oil-starved condition, more graphitic carbon was required to transfer onto the point of the opposite cast iron where no tribofilm occurred, an increasingly continuous carbon film formed over the residual tribofilm under the stressed shearing condition in Fig. 13. Meanwhile GLC surface should be more graphitic with good orientation toward the opposing surface in Figs 5 and 6.

**Figure 15:**
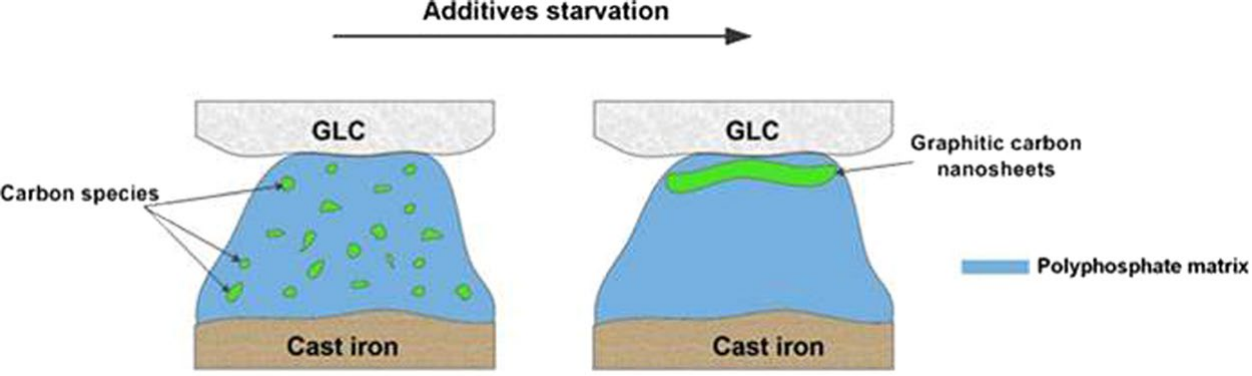
Schematic diagram showing the evolution of the tribofilm formed on GLC/Cast iron sliding couples as a result of lubricant starvation.

From a nanoscale viewpoint, amorphous carbon could create a planar orientation of carbon species within the reactive boundary layer^44, 45^. The adaptive contact therefore achieved the low shearing strength and helped improve the anti-friction characteristics and governed boundary lubrication. The application of GLC surface enables the running stability and friction reduction even if ZDDP tribofilm was not replenished timely. In this work, the GLC surface acted as a low friction layer which could compensate for loss of lubrication during extended sliding. Although the friction characteristics of this couple were low and stable (Fig. 1), wear between GLC/cast iron contacts slowly increased with sliding (Fig. 2). A clear step increase of specific wear rate of GLC was found after 8 h of sliding. The effect of poor lubrication on sliding was a structural reorganization and compositional variation of the tribofilm. This has been found in other mated surfaces involving viscoplastic contact or diffusion-dominated effects^46^. As a result, a secondary structure formed at the frictional interface in a partial pattern. In this work, diminishing lubrication resulted in carbon species combining with an alkaline composite (that acted as the binder) as shown in Fig. 11d. The transferred graphitic carbon film was found to cover the tribofilm in Fig. 13. The corresponding schematic showed the acting mechanism of GLC/cast iron contacts in Fig. 15.

## Conclusion

When CrN and CrN + GLC surfaces are slid against cast iron, wear and friction differed greatly. Tribofilms with different hierarchical structures and chemical compositions and distributions resulted. The sliding interface was understood in terms of the HSAB principle and through TEM/EDS observation. With the CrN/cast iron pair, increasing wear and friction occurred with diminishing lubrication. Within the tribofilm, sulfides form in the near-surface region and give rise to stable friction. In the case of GLC/Cast iron contact, the GLC surface further reduced friction and made the sliding much smoother when it was subjected to increasingly oil-starved conditions. The shearing pressure not only induced graphitization of GLC itself and also oriented carbon onto the opposing surface by the interfacial materials transfer. This helped maintain low friction and wear conditions even under oil-starved conditions. The combination of tribofilm from lubricant additives and a responsive formation of oriented carbon film at nanoscale were responsible for the performance in this study. GLC can work in extremely oil-starved condition and increase the durability of engine components. However, GLC itself suffered an acceleration of wear.

## Materials and Methods

### Materials

Two types of engineered non-ferrous surfaces on piston rings were used in this study; the coatings were PVD-CrN and PVD-CrN + GLC. A detailed deposition protocol for PVD-CrN and PVD-CrN + GLC is available in ref. 18. A counterpart has been obtained from the boron alloy cast iron cylinder liner. Some parameters of non-ferrous surfaces and cast iron counterpart are summarised in Table 3.

**Table 3:** Properties of coatings and cast iron counterparts.

Parameters	PVD-CrN	PVD-CrN + GLC	Boron alloy cast iron
Deposition method	Arc deposition	Arc + Magnetron sputtering	—
Hardness (HV50g)	1000–1200HV	1100–1300HV	400 ± 50HV
Surface roughness (R_a_, nm)	284	150	73
Coating thickness (μm)	37	40	—

Formulated 15W-40 CF-4 diesel oil from the Great Wall Lubricant Corporation of China was used to lubricate the piston ring/cylinder liner pairs; its viscosity index was 139, and the kinematic viscosity is 112.8 mm^2^/S at 40 °C and 15.28 mm^2^/S at 100 °C, respectively; this commercial formulation included ZDDP additives.

### Wear and Friction Tests

Prior to the frictional testing, the samples were cleaned with acetone in an ultrasonic bath for 15 mins to remove contaminants. The friction and wear of the contacts was evaluated with a High Frequency Oscillating Reciprocating Tribotester (SRV-IV, Optimal, Germany) under lubricated conditions. In the Ring-on-Block configuration, a coated piston ring (10 cm long and a conformal radius of 4.6 cm) formed the upper running part and the lower stationary block was from a boron alloy cast iron cut from the bulk cylinder (3 × 1 × 0.5 cm^3^).

The friction tests involved a running-in and a steady state period. A 50 N load is applied for a 20 min running-in period and then increased to 300 N, which is equivalent to a maximum linear contact pressure of 428571 N/m, for the steady period. A linear speed of 0.12 m/s was maintained throughout these periods at a reciprocating stroke of 2mm and a frequency of 30 Hz. The working temperature was kept at 170 °C. Prior to each run, a 0.0285 ml dose of engine oil was injected onto the contact area with a micro-syringe. There are three repetitive measurements for each type of ring and cylinder liner, and the coefficients of friction and sliding time are recorded automatically during the test.

### Characterization

Prior to testing, the surface roughness of the samples was measured with a MicroXAM 3D non-contact surface mapping profiler; this profiler was also used to measure the amount of material lost from the moving parts. After the frictional tests, the worn morphologies were examined by scanning electron microscopy (JEOL JSM T330A) using a Energy Dispersive X-ray Spectroscopy (EDS). TEM (JEOL JEM-ARM200F aberration-corrected Scanning Transmission Electron Microscope, TEM) with Electron Diffraction and Electron Dispersive Spectroscopy (EDS) were utilised to assess the film thickness, visualise the nanostructure, and identify the crystalline phase within the tribofilm. The TEM samples were prepared using an FEI Nova 200 Dual-beam Focused Ion Beam (FIB) system with a Ga source and a fine beam current of 100pA to extract a cross-sectional sample within the wear track of the specimen (15 μm × 15 μm). Prior to the FIB lift-out procedure, a thin layer of Au was sputtered onto the wear track to protect the surface structure. HR800 Raman Spectrum (Jobin Yvon, France) was used to track the structural change of transferring carbon onto the surface of the cylinder and GLC onto the rings after friction, using an Ar^+^ laser of 532 nm and a resolution of 1 cm^−1^.

### Wear evaluation

The wear loss of rings is calculated according to the ref. 19 The loss of wear volume of the cylinder liner is calculated from the profiles of the wear tack as seen by a non-contact 3D surface profiler (model MicroMAXTM, made by ADE Phase Shift, Tucson, AZ, USA).

### Lubrication state calculation

The theoretical minimum film thickness (*h*_min_) and dimensionless lambda (Λ) ratio are calculated according to the empirical formulae proposed by Dowson-Higginson, as shown in the Equations (1) and (2)^47^, where the piston ring maintains a line contact with the surface of the opposite cylinder.

1 $${h}_{min}=2.65[{\alpha }^{0.54}\cdot {({\eta }_{o}\cdot {\bar{\rm u}})}^{0.7}\cdot {R}^{0.43}/({E}^{0.03}\cdot {W}^{0.13})$$

2 $${\rm{\Lambda }}=\frac{{h}_{min}}{\sqrt{{{R}_{q,a}}^{2}+{{R}_{q,b}}^{2}}}\,$$

where *R* is the ring radius (0.046 m),$\,{\eta }_{o}$ the dynamic viscosity of oil (4.23 × 10^−3^ N·s/m^2^), *E* the overall elastic modulus of ring/cylinder contact (193 × 10^9^ N/m^2^), ${\bar{\rm u}}$ the linear sliding speed (0.12 m/s), *W* the contacting load (428571 N/m), *α* the pressure-viscosity coefficient of oil (2.2 × 10^−8^ N/m^2^). The minimum film thickness (*h*_min_) is 21.7 nm, and the lambda ratio (Λ) is 0.13 for GLC/cast iron and 0.073 for CrN/cast iron, respectively. Boundary lubrication was therefore prevalent during this frictional testing.

## Electronic supplementary material

Supplementary Information
