# Supplementary material for: Tribochemistry of adaptive integrated interfaces at boundary lubricated contacts
Source: Sci Rep. 2017 Aug 30;7:9935. doi: 10.1038/s41598-017-09879-7 (PMC5577201; doi:10.1038/s41598-017-09879-7)
Supplement: Supplementary file 1 — Supplementary Information [file 41598_2017_9879_MOESM1_ESM.pdf]

Title: Tribochemistry of adaptive integrated interface at boundary lubricated contacts

Shanhong Wan[1], Anh Kiet Tieu[1], Yana Xia[1], Liping Wang[2], Dongshan Li[2], Guang'an Zhang[2], Hongtao Zhu[1], Bach H. Tran[1], David R. G. Mitchell[3]

[1]*Faculty of Engineering and Information Sciences, University of Wollongong, Wollongong 2500, Australia*

[2]State Key Laboratory of Solid Lubrication, Lanzhou Institute of Chemical Physics, Chinese Academy of Sciences, Lanzhou 730000, China

[3]Electron Microscopy Centre, University of Wollongong, Wollongong, NSW 2522, Australia

Email: ktieu@uow.edu.au, lpwang@licp.cas.cn

1. Chemical Composition Analysis on the Sliding Surfaces by EDS

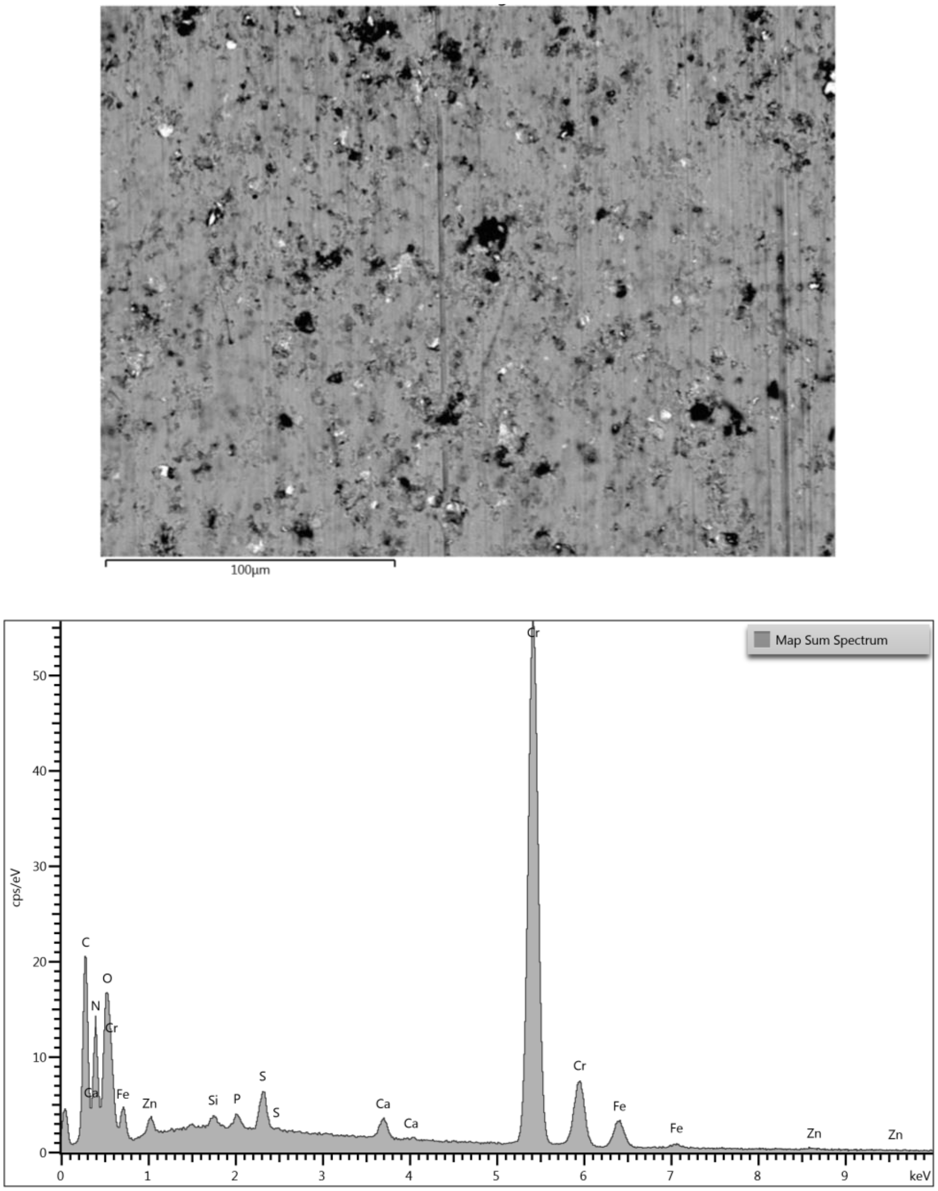

Figure S1 EDS analysis on the worn CrN surface after 4-hour sliding

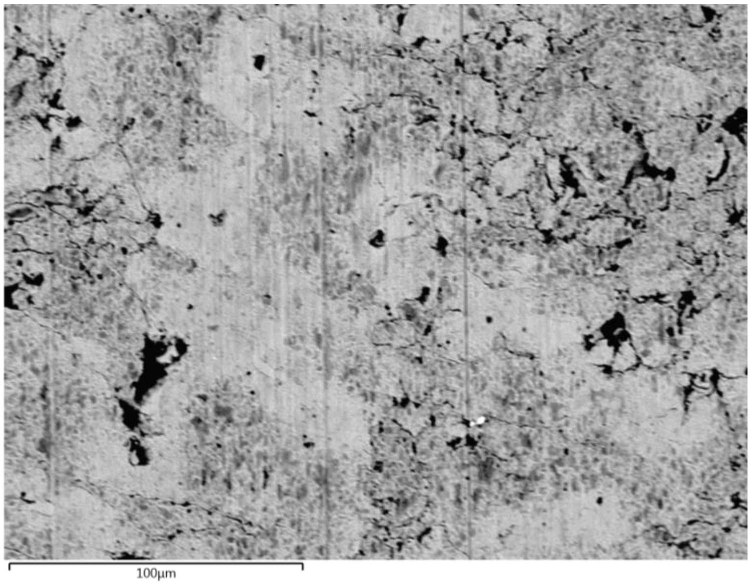

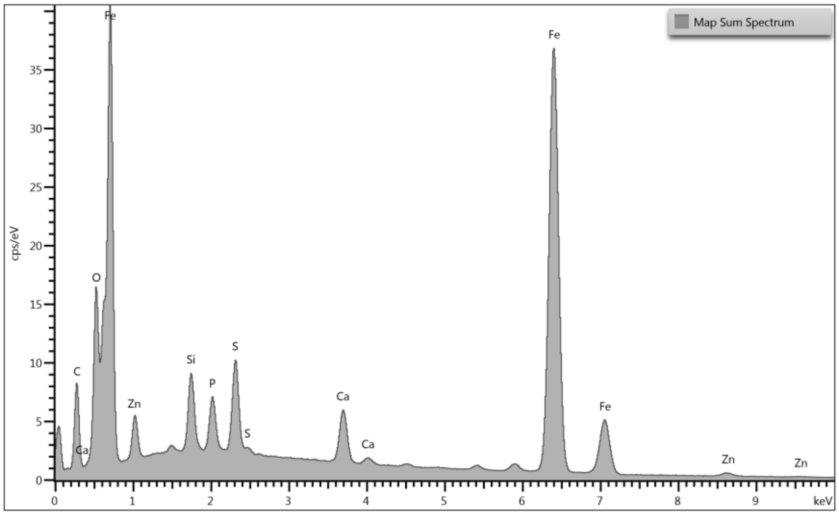

Figure S2 EDS analysis on the worn cast iron surface against CrN after 4-hour sliding

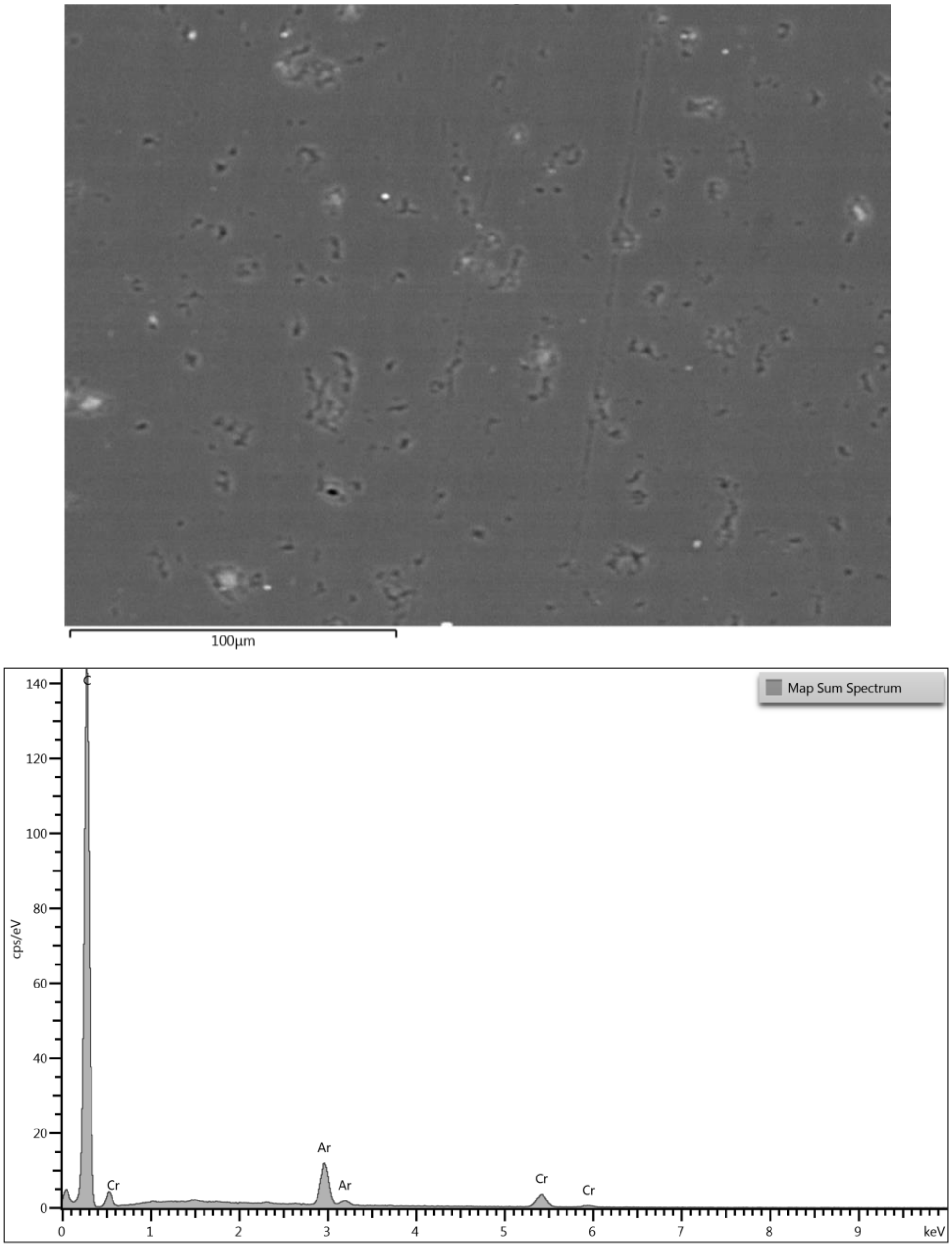

Figure S3 EDS analysis on the worn CrN+GLC surface after 4-hour sliding

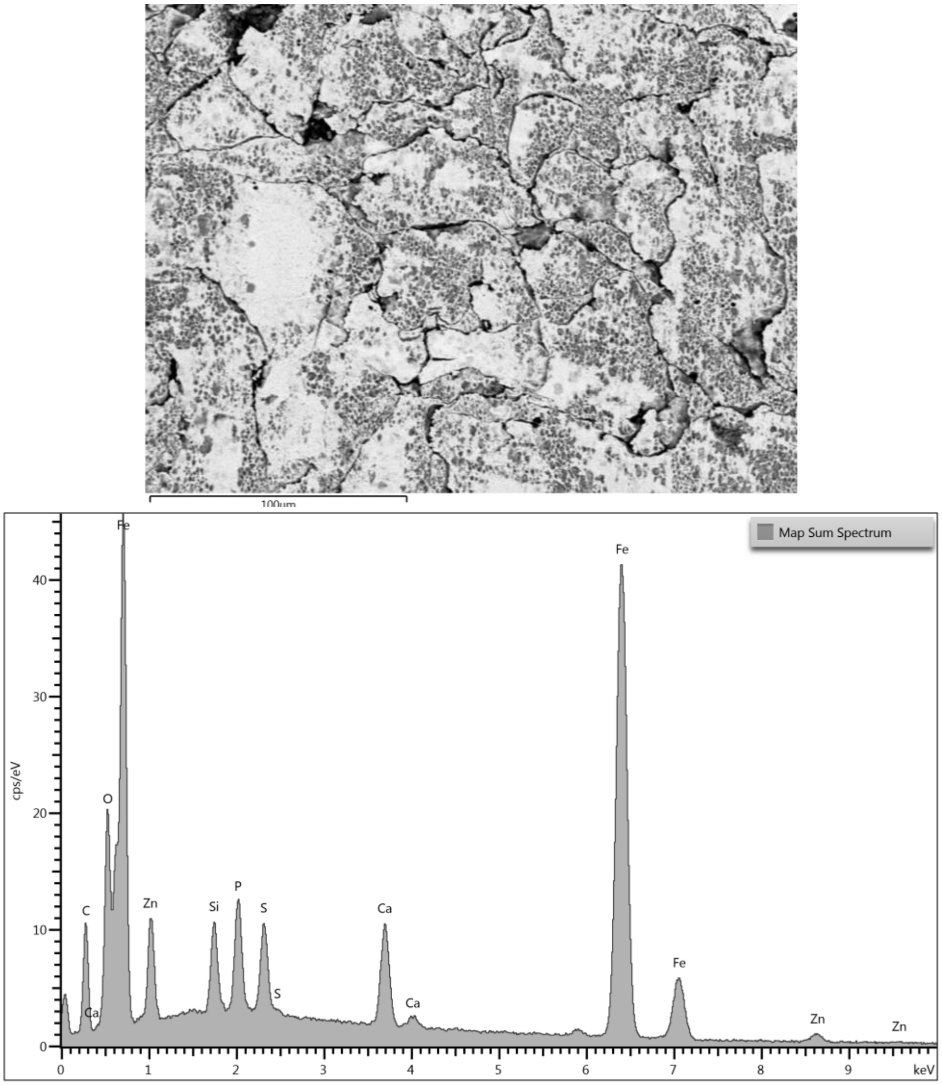

Figure S4 EDS analysis on the worn CrN+GLC surface after 4-hour sliding

2. Raman Analysis on the CrN/cast iron Contact

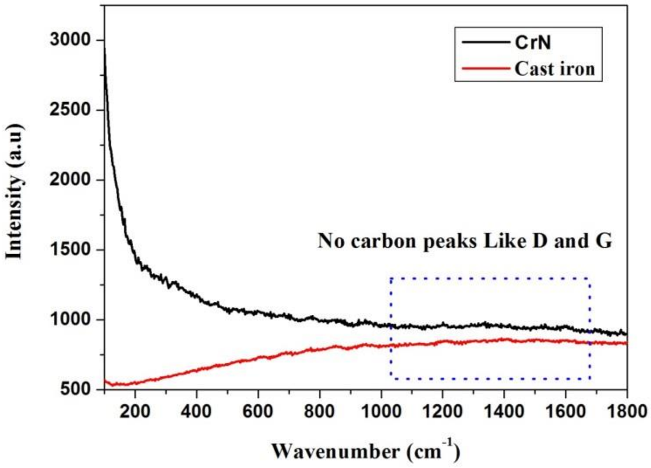

Figure S5 Raman analysis on the CrN/cast iron contacts after 4-hour sliding

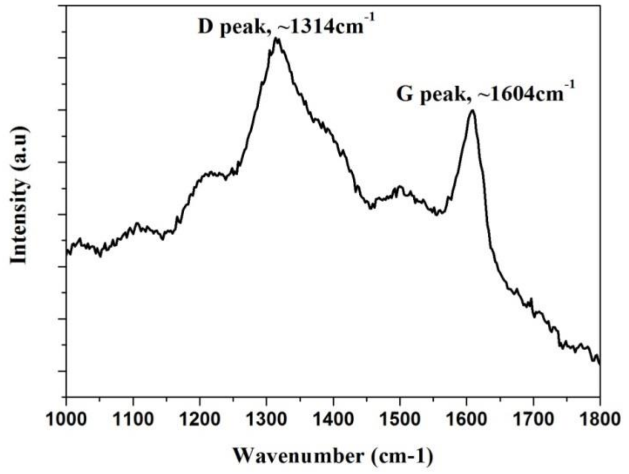

Figure S6 Raman analysis of graphite flakes within cast iron

Figure S1 and Figure S2 show the EDS analysis on the worn CrN and corresponding cast iron surface after 4-hour sliding, respectively. Zn, P, S, P and Ca elements can be detected in Figure S1 and Figure S2, while Si, Cr and Fe elements are from the mated solid contacts. Figure S3 and Figure S4 show the EDS analysis on the worn CrN+GLC and corresponding cast iron surface after 4-hour sliding, respectively. There is nothing relevant to the composition of lubricant additives on the worn GLC surface in Figure S3, while Zn, P, S, P and Ca elements can be identified on the corresponding cast iron surface in Figure S4. Figure S5 confirms no carbonaceous species growing on the worn CrN/cast iron contacts. Figure S6 shows the graphite flakes in cast iron.

3. EDS analysis

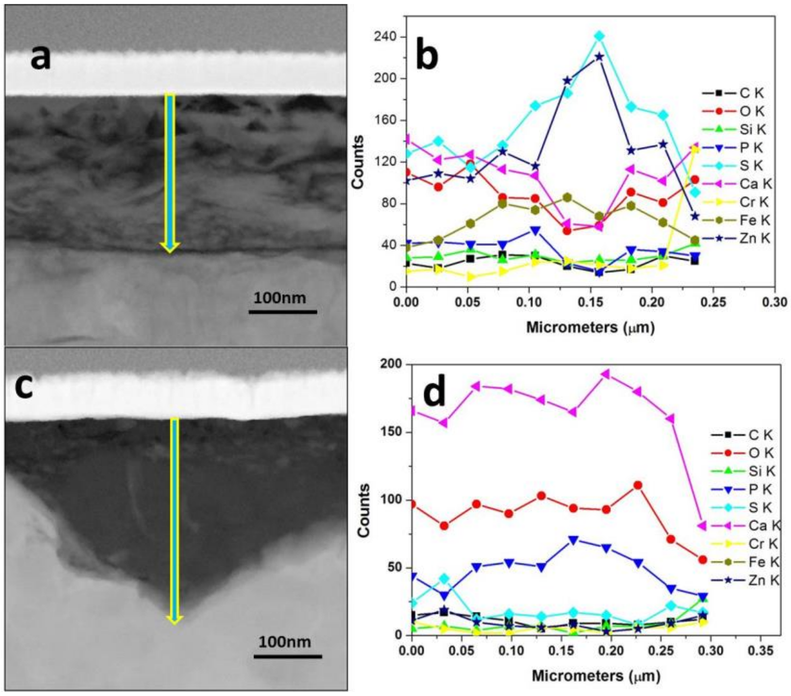

Figure S7 Cross-sectional view and linear element distribution of tribofilm on the CrN (a, b) and cast iron (c, d) surfaces after 1-hour sliding

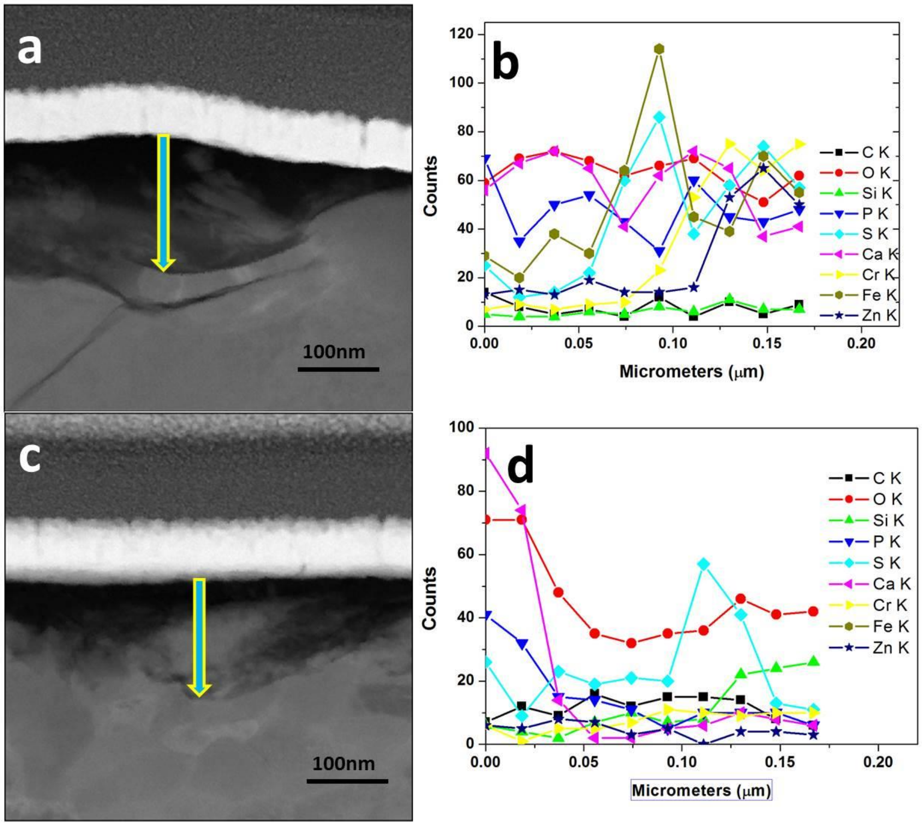

Figure S8 Cross-sectional view and linear element distribution of tribofilm on the CrN (a, b) and cast iron (c, d) surfaces after 8-hour sliding

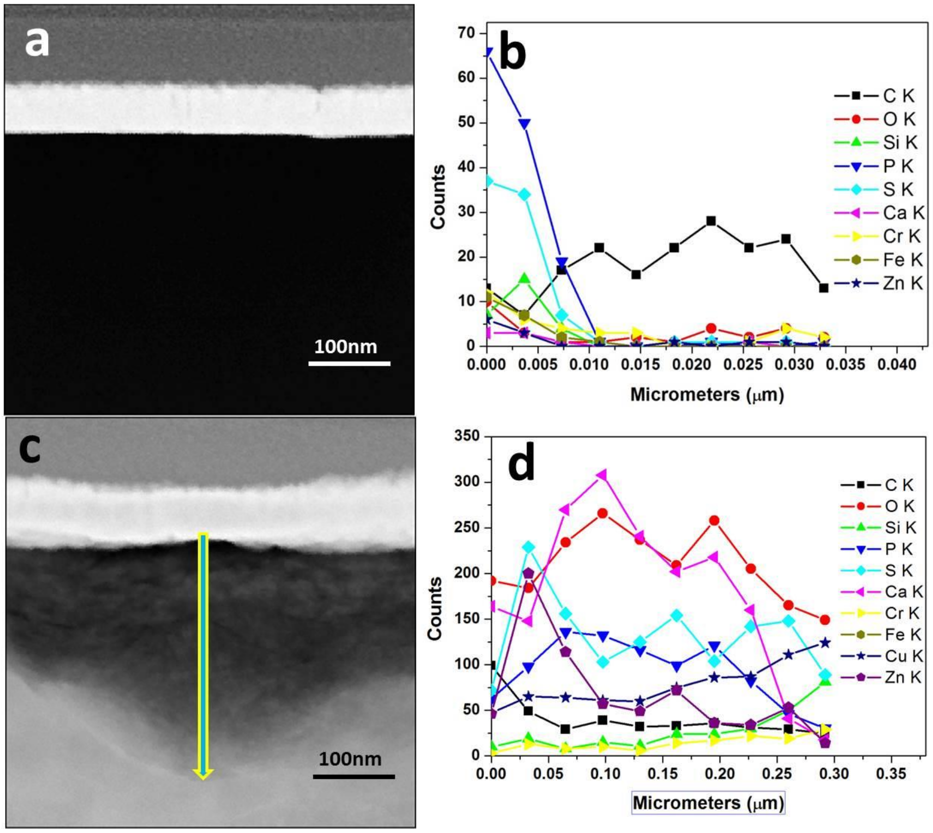

Figure S9 Cross-sectional view and linear element distribution of tribofilm on the CrN+GLC (a, b) and cast iron (c, d) surfaces after 1-hour sliding

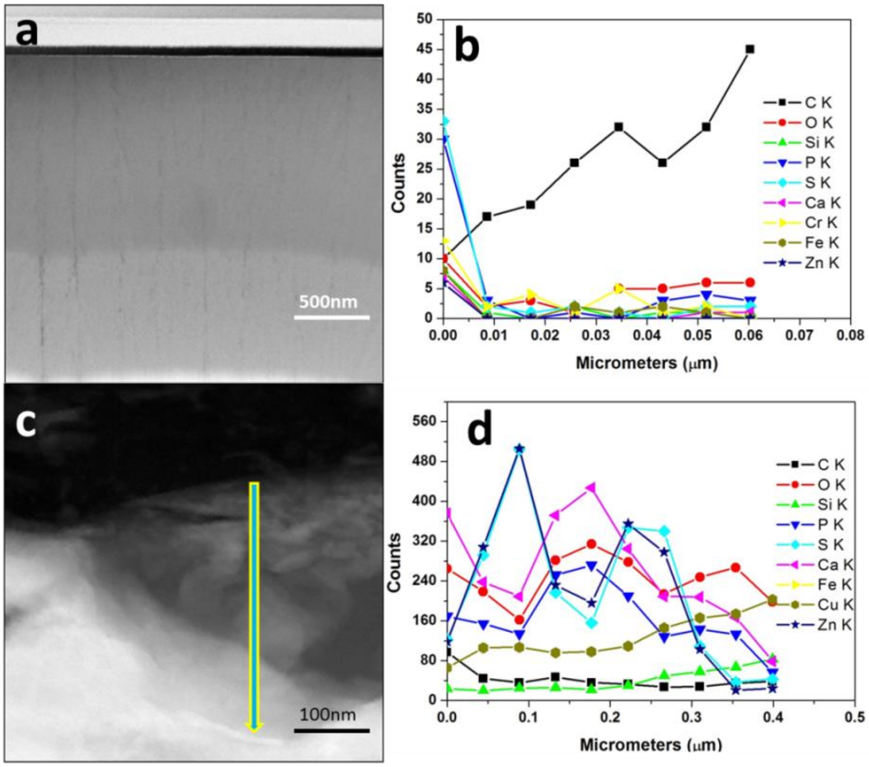

Figure S10 Cross-sectional view and linear element distribution of tribofilm on the CrN+GLC (a, b) and cast iron (c, d) surfaces after 8-hour sliding
